# Surgical versus non‑surgical treatment of proximal humerus fracture in patients aged 50–65 years: young shoulder CARE (displaCed proximAl humeRus fracturE) trial—a pragmatic randomized controlled trial study protocol

**DOI:** 10.1186/s13063-025-09283-x

**Published:** 2025-12-29

**Authors:** Line Houkjær, Antti P. Launonen, Bakir O. Sumrein, Laura Kärnä, Zaid Issa, Kenneth Brian Holtz, Stig Brorson

**Affiliations:** 1 Centre for Evidence-Based Orthopaedics, Zealand University Hospital, Køge, Denmark; 2 Faculty of Medicine and Health Technology, Tampere University Hospital, Tampere, Finland; 3 Department of Clinical Medicine, University of Copenhagen, Copenhagen, Denmark

**Keywords:** Shoulder fractures, Patient-reported outcome measures, Middle aged, Orthopedic procedures, Outcome assessment

## Abstract

**Background:**

Proximal humerus fractures (PHF) are common in adults above 50 years, often following low-energy trauma and underlying osteoporosis. Randomized trials in patients 60 years and older have found no clinically important difference in 1- and 2-year outcomes between surgical and non-surgical treatments. There is limited evidence for the 50–65 age group, who may have different functional demands and even a different overall health status. There is limited knowledge to guide treatment decisions for this age group.

**Method:**

A multicenter, parallel-group, randomized, superiority trial comparing osteosynthesis with non-surgical treatment in patients aged 50–65 years with displaced PHF. A total of 60 patients will be randomized equally to surgical treatment (primary osteosynthesis) or non-surgical treatment. Follow-up visits are arranged at 6 weeks and 6 months for all patients, with an additional 12-week visit mandatory for the surgical group and optional for the non-surgical group. The primary outcome is the between-group difference in Oxford Shoulder Score at 12 months. Sample size was calculated assuming a clinically relevant difference of 9.6 points on the OSS (SD 12), corresponding to 80% power at a 5% significance level. In case of treatment failure (persistent pain or a failed osteosynthesis), a salvage procedure will be offered. Patients not consenting to randomization will be asked to participate in a non-randomized cohort. They will have baseline data and preferences recorded, and they will receive the same follow-up and outcome assessments.

**Discussion:**

Young shoulder CARE trial aims to provide insights into the treatment of displaced proximal humerus fractures in patients aged 50–65 years, and to understand the benefits and harms of both surgical and non-surgical treatment options. The trial results will be published in an open-access peer-reviewed journal.

**Trial registration:**

Clinicaltrials.gov, NCT06416618, registered 14 May 2024.

**Supplementary Information:**

The online version contains supplementary material available at 10.1186/s13063-025-09283-x.

## Background

Proximal humerus fractures (PHF) are common injuries with an incidence of 138 per 100,000 person-years in adults aged 18 years and above in Denmark, increasing with age and have remained stable over decades [1]. In Denmark (2018), the total annual number of PHF was 6318 (72% women) [1]. In Finland (2019), the incidence among individuals aged over 16 years was 105 per 100,000. The incidence was higher in females and increased with age, particularly among those aged 50 years and older. The total annual number in 2019 was 4826 cases [2]. Less than half of the fractures are minimally displaced [3, 4], with displaced fractures defined as at least one of Codman’s four anatomic segments being displaced by ≥ 1 cm or angulated ≥ 45° (as defined by Neer [5]).

Meta-analyses comparing surgical and non-surgical treatments for PHF have been rated as having critically low methodological quality [6]. A Cochrane review reported high‐certainty evidence of no clinically important difference between surgical and non‐surgical treatment in patient‐reported shoulder function 1 and 2 years after injury [7]. Most included studies focus on older patients; however, a subgroup analysis (aged 16–65) from the ProFHER study [8] found no statistically significant differences in patient-reported shoulder function (Oxford Shoulder Score (OSS)) between surgical and non-surgical treatment at 6 months, 1 year, and 2 years. There was a trend toward more rapid improvement among younger patients (< 65 years) in the non-surgical group compared to those in the surgical group.

This study focuses on patients aged 50–65. Individuals in this age group often differ from older adults in terms of functional demands, work status, activity level, overall health, and rehabilitation expectations. PHF is uncommon in patients below the age of 50, whereas incidence increases markedly from this age and onward [1, 2]. More than 80% of the fractures occurred in patients above 50 years [6], in females [1, 2, 9] and results from low-energy trauma [3, 9]. Around this age, hormonal and metabolic changes begin to influence bone quality turnover [10] and may effect fracture healing [11]. Stratification by sex reveals a female predominance from the age of 50 [1, 2, 6], which coincides with an increase in age-related decrease of bone mineral density [10]. The exact prevalence of osteoporosis in the general population is not precisely known, as detection often relies on bone mineral density measurements, which are not routinely performed. Reduced bone mineral density is a known risk factor for complications following osteosynthesis [12].

The surgical treatment of choice for displaced PHF in adults 50–65 years is anatomical reduction followed by osteosynthesis if possible [13–15]. Surgical options include internal fixation with locking plates, intramedullary nails or joint replacement with hemiarthroplasty, or reverse shoulder arthroplasty. Complication and reoperation rates are high after surgery [16].

Orthopedic surgeons may encounter challenges in making treatment decisions with patients in this specific age group, as existing randomized trials have predominantly focused on older individuals [17]. There is an intermediate age group of patients for whom it remains uncertain whether surgical intervention is superior to non-surgical management in terms of benefits and harms.

The aim of this study is to assess whether osteosynthesis results in superior patient-reported shoulder function compared to non-surgical treatment, as measured by the Oxford Shoulder Score at 12 months, in patients aged 50 to 65 years with displaced proximal humerus fractures.

We hypothesize that surgical treatment with osteosynthesis results in superior patient-reported shoulder function, measured by the Oxford Shoulder Score at 12 months, compared to non-surgical management in patients aged 50–65 years with displaced proximal humerus fractures. Accordingly, the null hypothesis states that shoulder function, measured by the Oxford Shoulder Score, is not superior following surgical compared to non-surgical treatment.

## Methods: patients, intervention, and outcomes

### Study design

The study is a two-center, parallel-group, randomized, superiority trial comparing osteosynthesis with non-surgical treatment following a displaced PHF in patients aged 50–65 years with a 1:1 allocation ratio.

The protocol will follow the PREPARE trial guide [18] and the SPIRIT checklist [19]. PRECIS-2 assessment indicates that the trial is pragmatic with 42 points out of 45 points (see Supplementary information, file 1) [20]. The study will be reported according to the CONSORT checklist [21]. The statistical analysis plan (Supplementary information, file 2) will follow the Guidelines for the Content of Statistical Analysis Plans in Clinical Trials [22]. All checklists are available in Supplementary information, file 3.

**Fig. 1:**
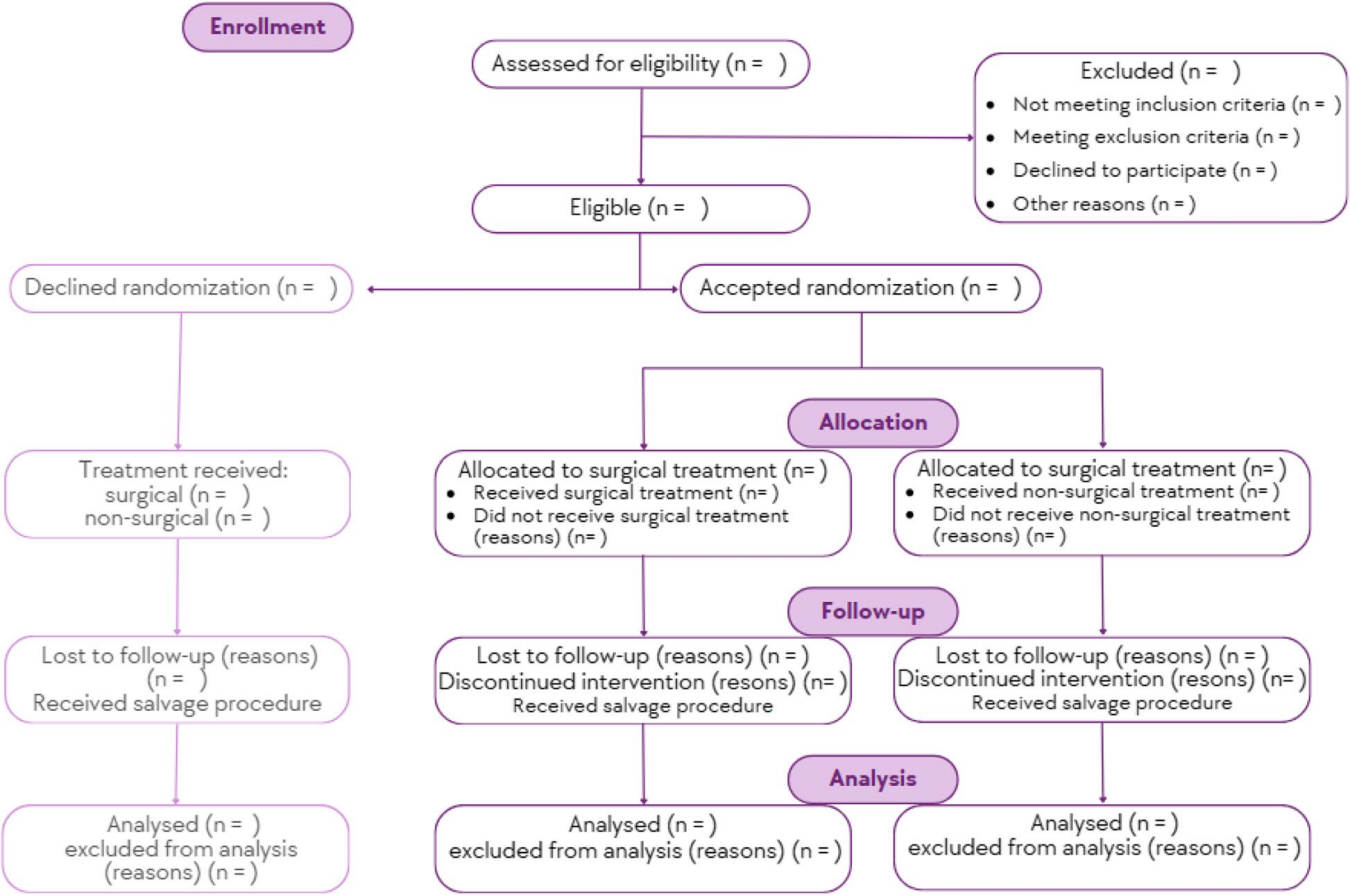
Trial flowchart

The trial flow and timeline for data collection are outlined in Fig. 1 and Table 1.

**Table 1:** Timeline and overview of enrolment, intervention and assessments

		Study period							
	Enrolment	Allocation	Post-allocation						Close-out
Timepoint	Pre-randomization	Randomization	Day of intervention (2 weeks post-injury)	Week 2^a^	Week 6^a^	Week 12^a^	6 months^b^	12 months^b^	24 months ^b^
Enrolment									
Eligibility screen	x								
Informed consent	x								
Allocation		x							
Intervention									
Surgical treatment			x						
Non-surgical treatment			x						
Assessments									
Radiographs, surgery	x		x	x		x	x		
Clinical control surgery			x	x		x	x		
Radiographs, non-surgical	x		x		x	(x)^c^	x		
Clinical control, non-surgical			x		x	(x)^c^	x		
Referred to DXA-scan			x						
OSS							x	x	x
EQ-5D-3L							x	x	x

^a^For surgery-group, number of weeks after surgery. For the non-surgical group, number of weeks after injury

^b^Number of months post injury, regardless of group

^c^Possible additional control

### Participants

Patients will be recruited from the orthopedic departments at Zealand University Hospital in Denmark and Tampere University Hospital in Finland.

The following inclusion criteria will apply:

- Patients aged between 50 and 65 years with a displaced proximal humerus fracture after a low-energy trauma.
- Patients should be cognitively capable of answering the follow-up questionnaires.

The following exclusion criteria will apply:

Communication:

- The patient does not understand written and spoken native language (Danish or Finnish)
- Inability to give informed consent

Fracture criteria:

- Fractures assessed to be non-reconstructable by the treating surgeon
- Isolated tuberosity fracture, fracture dislocations, open fractures, and fractures with involvement of the articular surface
- Less than 25% contact between the humeral head and humeral shaft measured at two perpendicular radiographs at 2 weeks postinjury
- Pathological fractures or previous fractures in the same proximal humerus
- Concomitant fractures, which could influence the outcome

Other criteria:

- Paralysis of the upper extremity

### Recruitment

Patients admitted to the emergency department will be clinically examined by the emergency doctor. Two perpendicular plain radiographs will be obtained. If a displaced PHF is diagnosed, the patient will be referred to the outpatient clinic within 2 weeks postinjury. Patients will be screened for eligibility based on the initial radiographs and medical records by the investigator at the clinic. During the first visit to the outpatient clinic, the patient will be informed about the study. Potential participants will receive verbal and written information about the study from the local investigator. If eligible and consenting to participate, the local investigator will provide face-to-face trial instructions, and informed consent will be obtained.

### Non-randomized cohort

Eligible patients who decline participation will be recorded; when possible, their reasons for declining will be noted. These patients will be invited to participate in the non-randomized cohort. The patient can choose surgery only if recommended by the surgeon; such cases will be reported. Patients in the non-randomized cohort who provide informed consent will participate in the same treatment, rehabilitation, follow-up, and outcome assessments as the randomized cohort, with data recorded for research purposes.

### Randomization and concealment of allocation

We will use Research Electronic Data Capture (REDCap) [23] to generate an irreversible randomization sequence. A person not involved in the project will generate and upload the allocation table using “R.” All investigators will be blinded to the allocation table. The person not involved in the project will have no access, including no access to the data. We will be using block randomization with varying block sizes of 4 and 6, stratified by location. REDCap will conceal the sequence for the investigators before allocation takes place. Enrolment and allocation will occur on-site by LH, AL, BS, or LK. A REDCap built-in functionality ensures that only patients who meet all inclusion and no exclusion criteria can be randomized.

### Intervention

All patients will receive standard pain management following local guidelines and will be provided with a sling and swathe on the day of the injury in the emergency room. After 10 to 14 days, all patients will undergo evaluation in the outpatient clinic.

#### Surgical group

The surgical group will receive osteosynthesis with a locking plate (Philos System, J&J DePuy Synthes) or an intramedullary locking nail (Multiloc Humeral Nail, J&J DePuy Synthes), according to the surgeon’s preference. The patients will be provided with a sling and swathe immediately after the surgery. From the first postoperative day, only the sling will be used. Two weeks postoperatively, patients will undergo clinical and radiological follow-up in the outpatient clinic. At 12 weeks postoperatively, patients will have another clinical and radiological follow-up. This is the current standard care for surgical treatment with a plate or intramedullary nail. All surgeries in this study will be performed by specialized upper limb trauma or shoulder surgeons, each with a minimum of 5 years of subspecialty experience.

#### Non-surgical group

Patients in the non-surgical group will be offered an optional sling for comfort for an additional 1–2 weeks at their first visit, 10–14 days post-injury. At week 6 post-injury, all patients will undergo clinical and radiological follow-up. This represents the current standard care for non-surgical treatment of displaced PHF at the departments.

If there is still severe pain at week 6, it is considered a treatment failure. Severe pain is defined as being unable to sleep due to pain and/or requiring opioids to manage it (without opioid use prior to injury). If there is little progress but moderate pain, an additional consultation can be provided 12 weeks post-injury with clinical and radiological follow-up.

#### Both groups

Patients will be offered standard 6-month radiographs and clinical assessments, as well as a diagnostic workup for osteoporosis with a DXA scan. Osteopenia is defined as a *T*-score between − 2.5 and − 1. Osteoporosis is defined as a *T*-score lower than or equal to − 2.5 [24]. Additionally, osteoporosis can be diagnosed by hip and spine fractures, which, in Denmark, is considered pathognomonic for osteoporosis.

Boths groups will be referred to rehabilitation in the municipalities. An overview of the rehabilitation timeline is presented in Table 2. Patient information sheets are provided in Supplementary file 4. The rehabilitation program is similar, starting post-surgery for the surgical group and from the time of injury for the non-surgical group. The duration, intensity, and exercises used will be tailored in collaboration between the patients and the physiotherapist. Rehabilitation typically lasts 6–12 weeks. Adherence to rehabilitation is defined as completing at least 6 weeks of rehabilitation and following rehabilitation protocol.

**Table 2:** Rehabilitation timeline for surgical, non-surgical, and salvage groups

Group	Weeks after operation/injury	Rehabilitation activities
Surgical group	Week 0–6	Free hand, wrist, elbow, and cervical movement in a full range of motion daily is advised. Free movement exercises with a maximum of 1 kg load. Avoid outward rotation beyond the neutral position and no inward rotation corresponding to the hand behind the back. We recommend using the arm for daily activities as soon as the pain allows. Passive movement with a focus on mobility and gradually additional focus on active movement
	Week 6–12	Free movement exercises with gradually increased load
	From week 12	Full load allowed
Non-surgical group	Week 0–2	Sling and swathe worn at all times
	Week 2–6	Begin pendulum exercises with circles in and out. Free movement exercises except outward rotation beyond neutral position and no inward rotation corresponding to the hand behind the back. We recommend using the arm for daily activities as soon as the pain allows. Passive movement with a focus on mobility and gradually additional focus on active movement
	Week 6–12	Free movement exercises with gradually increased load
	From week 12	Full load allowed
Salvage procedure	Week 0–6	Free hand, wrist, elbow, and cervical movement in a full range of motion daily is advised. Free movement exercises with a maximum of 1 kg load. Avoid outward rotation beyond the neutral position and no inward rotation corresponding to the hand behind the back. We recommend using the arm for daily activities as soon as the pain allows. Passive movement with a focus on mobility and gradually additional focus on active movement
	Week 6–12	Free movement exercises with gradually increased load
	From week 12	Heavier load allowed (max. 15 kg/35 lbs lifelong)

#### Treatment failure

In case of treatment failure in either group, defined as persistent pain or a failed osteosynthesis, a salvage procedure with secondary osteosynthesis, including the possibility to use a locking plate with a graft or a reverse shoulder arthroplasty (RSA), will be offered. The decision to proceed to a salvage procedure will be made in collaboration with the patient and the surgeon. Failed osteosynthesis and the most appropriate salvage procedure will be defined by the surgeon.

Cross-over to RSA or revision osteosynthesis is considered a failure of treatment regardless of group allocation. The patient will remain in the study. The reason for crossing over will be noted and reported.

### Strategies to improve retention

Patients will receive a phone number to contact the local investigator with any questions. All included patients will be seen by either LH, SB, AL, BS, or LK at all appointments. Follow-up is planned at 6, 12, and 24 months. The 6-month follow-up includes radiographs, clinical assessment (range of motion, strength, and test for subacromial pain) and questionnaires. Data will be stored using Research Electronic Data Capture (REDCap) [23]. At the 12- and 24-month follow-ups, questionnaires will be automatically sent by REDCap for patients to complete from home, provided the patient has a mailing address. Patients are asked to accept telephone calls if they miss follow-up visits to promote patient retention and complete follow-up. Reminders will be sent 2 and 4 weeks after receiving their 12- and 24-month questionnaires. After 6 weeks, completion by phone will be offered if still unanswered [25].

### Outcome

The primary outcome will be the OSS at 12-months. The secondary outcome will be OSS at 6- and 24-month and EQ-5D-3L score measured at the 6, 12, and 24 months. Secondary outcomes will include the incidence of adverse events in all groups and the rate of conversion to surgery in nonoperative group.

#### Oxford Shoulder Score

OSS is a patient-reported shoulder-specific outcome measure to assess shoulder function. It consists of 12 questions on a 5-point Likert scale (both pain- and function-related), each offering five ordinal response options. The cumulative score ranges between 0 and 48, being 0 (worst) to 48 (best, no pain, or functional limitation). The questionnaire includes a mix of functional ability and pain-related questions. It is validated in adults with shoulder pain from a degenerative or inflammatory condition (median age 57.4 in the UK) [26], and in Denmark in patients with post-traumatic shoulder diseases, including impingement and sequelae after PHF [27]. Patients in follow-up cohorts do not need to attend the clinic in person to complete the OSS [27].

#### Quality of life

EQ-5D-3L is a generic health-related quality-of-life assessment tool validated as a quality-of-life measure in patients with PHF [28]. It consists of a 5-dimension descriptive questionnaire about mobility, self-care, usual activities, pain/discomfort, and anxiety/depression as a measure for health-related quality of life [29]. Each dimension has 3 levels: no problems, some/moderate problems, and unable/extreme problems. Each dimension is weighted differently and results in a total score based on the EQ-5D index calculator (TTO). The EQ-5D-3L also includes an overall scale (numeric rating scale) in which patient rates their overall health condition today on a scale between 1 and 100, with 100 being the highest level of health imaginable.

#### Adverse events

Serious adverse events, as defined by the WHO [30], will be reported to a data monitoring committee (DMC). These events are defined as those leading to death, being life-threatening, requiring inpatient hospitalization, or necessitating reoperation.

Other adverse events, whether surgical or non-surgical related, will also be reported.

Systematic reviews on terms and definitions for complications after surgical [31] and non-surgical treatment [32] have been conducted. Based on international consensus on the core event set for PHF, both surgical and non-surgical treatments, eight event groups are defined [33]. The following adverse event groups will be monitored: implant, osteochondral, shoulder instability, peripheral neurology, vascular, infection, device, superficial soft tissue, and deep soft tissue. In addition to this, we have chosen to monitor clinical symptoms such as persistent severe pain. The event groups are elucidated in Supplementary information, file 5 – Complications.

#### Conversion to surgery

The criteria for conversion to surgery are based on an overall clinical evaluation conducted in collaboration with the patient. In the non-surgical group, the primary reason for conversion is persistent severe pain after 6–12 weeks. In the surgery group, adverse events can serve as reasons for conversion to RSA. Patients who discontinue for reasons unrelated to surgery will be documented and recorded. Patients receiving osteosynthesis or RSA will also have follow-up assessments after 6, 12, and 24 months.

## Statistical methods

### Hypothesis

The null hypothesis is that the shoulder function measured with OSS 12 months after surgery is not superior to non-surgical treatment.

The alternative hypothesis is that the shoulder function measured with OSS 12 months after surgery is superior to non-surgical treatment.

### Sample size and power considerations

The standard deviation (SD) for OSS has been reported as 12 [34]. While the minimal clinically important difference (MCID) for the OSS (0–48) has been reported to range from 5 to 6.9 points in various shoulder diseases, age groups, and assessment points, it has not been established for PHF [35]. Without a patient-derived MCID for PHF, a relevant difference of 9.6 was assumed to represent a clinically meaningful difference. This corresponds to approximately a 20% difference between the surgical group and the non-surgical group on a 0–48 OSS scale. Based on this assumption, with a power of 80% and a 5% level of significance, a sample size of 25 patients per group is required. Assuming for a 15% potential loss to follow-up, the recruitment target is 30 patients in each group, resulting in a total of 60 patients. The recruitment period is expected to be 24 months.

### Statistical analysis

The statistical analysis plan is available within Supplementary Information, file 2.

Baseline characteristics will be presented using descriptive statistics. We will compare the following patient characteristics between the groups at baseline: age (categorized into 5-year age groups), sex, height, weight, ASA score, fracture classification (Neer [5]), dominant arm (yes/no), smoking, alcohol consumption, educational level, working status, work type, osteoporosis (three-level ordinal variable based on *T*-scores (osteoporosis, osteopenia, and normal bone density)), and clinical frailty scale [36]. SD will be reported, and *n* (%) for categorical values.

Osteoporosis will be collected and reported descriptively as a three-level ordinal variable based on *T*-scores (osteoporosis, osteopenia, and normal bone density).

The OSS results for surgical and non-surgical treatment groups at 6, 12, and 24 months will be tabulated and graphically represented with mean, SD, median and IQR according to the distribution.

The primary outcome, OSS at 12 months, will be analyzed using two-sample *t*-test comparing the two treatment groups. Normality assumptions will be assessed, and if violated, alternative methods such as transformation or non-parametric Mann–Whitney *U* test will be considered. As suggested in the extension of the CONSORT 2010 statement, the primary outcome will be analyzed for the intention-to-treat (ITT) population.

In addition, linear mixed models will be used to analyze OSS and EQ-5D over time (6, 12, and 24 months) accounting for the repeated measures structure and to explore outcome trajectories. Model-based contrasts from the LMM will be used to estimate adjusted between-group differences at specific time points. Missing data will be handled within the mixed model framework, assuming data are missing at random. Details are provided in Supplementary Information, file 2.

We will record and report adverse events in each group. At the 6-month follow-up, patients will be asked, and we will record and report the length of rehabilitation in municipalities (in weeks) and whether the patient has followed the rehabilitation plan.

#### Salvage procedure

Salvage procedures and adverse events will be reported descriptively. Reoperation in the form of hardware removal will be classified as a complication, not a salvage procedure. There will be two analyses for ITT: one including salvage procedures and one excluding them. The timing of salvage procedures will be analyzed using a Cox proportional hazards model to compare time to salvage between treatment groups.

#### Non-randomized cohort

Patients who declined randomization but consented to follow-up will form the non-randomized cohort. Baseline characteristics will be summarized descriptively and compared between the randomized and non-randomized cohorts, as well as among the non-randomized patients by treatment choice. The primary outcome (OSS at 12 months) will be analyzed by treatment modality. Secondary outcome measures (OSS and EQ-5D) will be presented descriptively and graphically at 6, 12, and 24 months. Analyses will include multivariable linear regression adjusted for baseline covariates; propensity score methods may be applied. The incidence and timing of salvage procedures will be described and analyzed using Cox proportional hazards models.

#### Blinded data interpretation

Although patients and investigators are not blinded to treatment allocation, the primary investigator will conduct blinded statistical analysis through data anonymization and will be supervised by a biostatistician. The OSS and EQ-5D-3L will be completed just before the 6-month visit, collected automatically through REDCap, and will not be reviewed by the investigator prior to analysis. After the blinded statistical analysis has been completed, two abstracts will be written based on the pre-blinding analysis results before the blinding is revealed.

Data will be analyzed using the statistical “R” [37].

#### Interim analyses

An interim analysis will be conducted when 50% (*n* = 30) of the randomized patients have completed their 6-month follow-up, focusing on OSS, failure to treatment, and serious complications. A third party will blind the data to treatment location before analysis. Regardless of whether the interim analyses suggest that the study is unlikely to achieve its primary objectives, the study will proceed as initially planned.

A DMC has been organized to monitor and evaluate the data from the interim analysis. The DMC consists of five independent members: two orthopedic researchers and an orthopedic nurse from Denmark, and one orthopedic researchers and one orthopedic nurse from Finland. All members have equal standing within the committee.

The DMC will monitor unexpected, unwanted events; continued inclusion will be considered if the failure rate is above 30%. Intervention-related hospitalization or mortality will be reported to the ethics committee.

### Data management

All study data will be collected and stored in Research Electronic Data Capture (REDCap) [23]. Clinical assessments and radiographs will be entered directly into REDCap by the investigators (LH, SB, AL, BS, or LK). Questionnaires at 12 and 24 months will be distributed electronically through REDCap. Data will be stored securely on institutional servers with access restricted to study personnel.

### Protocol violation

Patients who drop out of the trial will be noted, along with the reasons. The patients will remain in the study and be included in the ITT analysis. Numbers and time for loss to follow-up will be reported.

### Patient and public involvement

Four patients read and commented on the written information sheet, confirming the understandability and that it was sufficiently informative.

## Discussion

### Relevance of age in existing evidence

Current high-quality evidence on proximal humerus fracture treatment primarily concerns patients from 60 or above. This leaves a knowledge gap in the younger, still working-age population aged 50–65. A Cochrane review reported high- or moderate-certainty evidence that surgery, compared to non-surgical treatment after a displaced proximal humerus fracture, did not provide a better outcome after 1 and 2 years [7]. However, patients aged 50–65 may differ in functional demands, health status, and recovery expectations. Despite this, they are typically underrepresented in clinical trials.

The young shoulder CARE trial was designed to contribute to addressing this evidence gap. By focusing exclusively on patients aged 50–65, the trial aims to generate age-specific data to inform clinical decision-making for this population better. The pragmatic design was chosen to reflect real-world clinical practice and increase external validity.

### Choosing the age range

The age of 50 was chosen because PHF is uncommon in patients under 50, but its incidence increases significantly after this age [1, 2]. Over 80% of fractures occur in patients aged 50 or older [6]. Additionally, at Zealand University Hospital, patients aged 50 and older are routinely referred to a Fracture Liaison Service (FLS) for bone health assessment, supporting the choice of 50 years as a meaningful lower age limit for inclusion.

There is no universally agreed-upon age cut-off for what constitutes an “older” adult, yet age definitions are crucial in trial inclusion and generalizability. The WHO previously characterized older people as those over 65 years (2010 report), the latest age report has no precise definition. The term “older” is too complex due to multiple factors and variations in global age distribution, making it challenging to establish an age cutoff [38]. The Danish Health Authority [39] and International Population Report of the National Institutes of Health [40] define “older” as individuals surpassing the age of 65. In orthopedic research, Sabharwal found that age 65 is the most common age at which a person is considered “older” [41].

We aimed to investigate a population that differs from older adults in terms of functional demands, general health status, and expectations for recovery. To ensure the inclusion of individuals who are still active in the workforce, we focused on patients below the typical retirement age. In Finland and Denmark, the retirement age is 65–67 years [42, 43]. Consequently, we selected 65 years as the upper age limit to target a relatively younger and more active patient group. This allows our trial to clearly complement and extend existing evidence focused on older adults.

### MCID

Currently, an MCID for the OSS in patients with PHF has not been determined. In other shoulder conditions, the MCID for the OSS (ranging from 0 to 48) has been reported to be between 5 and 6.9 [35]. Nonetheless, the concept of MCID is inherently context-dependent and can vary based on factors such as age, sex, baseline functional status, injury mechanism, and comorbidities, and may also evolve over time [44]. Consequently, the MCID should not be regarded as a universal fixed value. In the absence of a PHF-specific MCID, this study adopted a difference of 9.6 points on the OSS—equivalent to 20% of the scale—as a clinically meaningful threshold. This decision informed the sample size calculation, under the premise that surgical intervention should produce a substantial benefit to be deemed appropriate. Future investigations should focus on validating or refining the MCID specific to PHFs.

### Limitations

Several considerations should be noted when interpreting the results of this study. First, only two centers were included, which may limit the generalizability of the findings to broader populations. This choice was deliberate, prioritizing centers where we could ensure complete follow-up and standardized screening of all eligible patients, allowing for consistent and reliable inclusion of the target population.

Second, the sample size is relatively small, and the study was not blinded, which is inherent in surgical trials, as both the surgeon and the patient are aware of the treatment received. To minimize bias related to outcome assessment, we chose patient-reported outcome measures (OSS), allowing patients to evaluate their own shoulder function directly. This approach avoids potential observer bias associated with surgeon-administered instruments.

Despite these limitations, the trial provides valuable age-specific data on patients aged 50–65, a group underrepresented in previous studies. These findings may inform future clinical decision-making and help refine treatment guidelines for displaced proximal humerus fractures in this intermediate age group, particularly regarding the choice between surgical and non-surgical management.

## Supplementary Information

Supplementary Material 1. File 1. PRECIS-2 assessment. File 2. Statistical Analysis Plan. File 3. SPIRIT CONSORT and SAP Checklist. File 4. Rehabilitation patient information. File 5. Complications [45]. File 6. Ethical approval. File 7. Salary guarantees and funding documentation

Supplementary Material 2

## Data Availability

Data sharing is not applicable to this article as no datasets were generated or analyzed during the current study. The Data Protection Agency of Region Zealand has approved the handling of personal data (p-2024–16591). Compliance with the General Data Protection Regulation (GDPR) and the Danish Data Protection Act will be ensured at all times. A data processing agreement has been signed in both recruiting centers. Research data is stored on REDCap, a secure online patient management program, accessible only to LH, SB, AL, BS, and LK on a secure server at Region Zealand University Hospital (with two-factor authentication login). The primary investigator will have access to the final data set. The REDCap setup ensures that randomization is only possible, if all baseline information is entered correctly. All data will be fully anonymized before publication. The trial results will be published in an open-access peer-reviewed journal.

## Abbreviations

ASA: American Society of Anesthesiologists Score
DXA: Dual-energy X-ray absorptiometry scan
EQ-5D-3L: European Quality of Life – 5 dimensions – 3 levels
ITT: Intention to treat
MCID: Minimal clinically important difference
OSS: Oxford Should Score
PHF: Proximal humerus fracture
PROM: Patient-reported outcome measure
RCT: Randomized controlled trial
REDCap: Research Electronic Data Capture
SD: Standard deviations
SPIRIT: The Standard Protocol Items: Recommendations for Interventional Trials
WHO: World Health Organization
