# Supplementary material for: Surgical versus non‑surgical treatment of proximal humerus fracture in patients aged 50–65 years: young shoulder CARE (displaCed proximAl humeRus fracturE) trial—a pragmatic randomized controlled trial study protocol
Source: Trials. 2025 Dec 29;26:588. doi: 10.1186/s13063-025-09283-x (PMC12752399; doi:10.1186/s13063-025-09283-x)
Supplement: Supplementary file 1 — Supplementary Material 1. File 1. PRECIS-2 assessment. File 2. Statistical Analysis Plan. File 3. SPIRIT CONSORT and SAP Checklist. File 4. Rehabilitation patient information. File 5. Complications [45]. File 6. Ethical approval. File 7. Salary guarantees and funding documentation [file 13063_2025_9283_MOESM1_ESM.pdf]

Supplementary Information, File 1

PRECIS-2 wheel

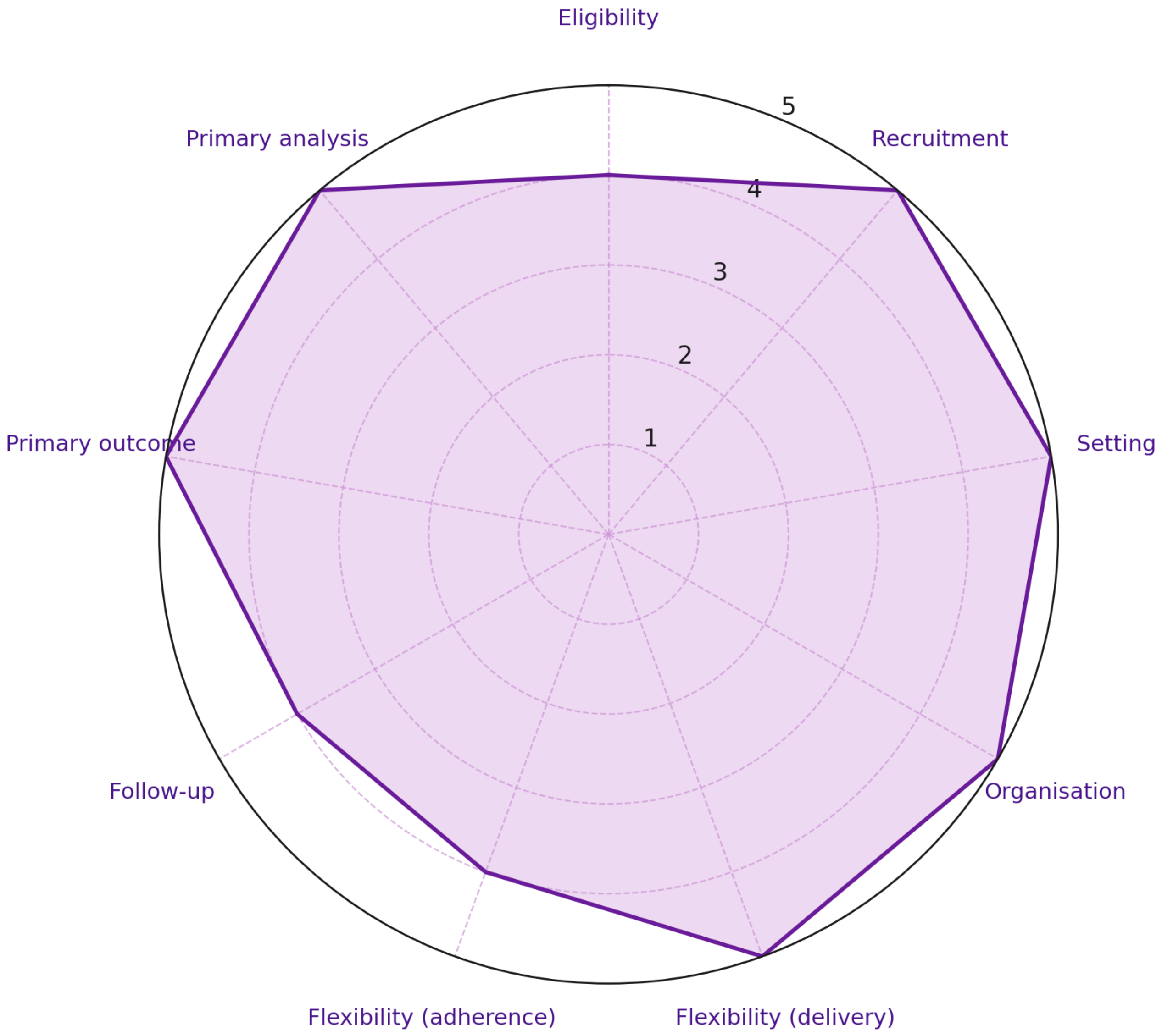

PRECIS-2 scores for trial domains

Domain	***Score***	***Rationale***
Eligibility	4	Rather pragmatic: Inclusion criteria are broad (50–65 years, displaced PHF, cognitively able), excluding only non-reconstructable or complex fractures, or paralysis of the upper extremity . This allows generalizability to typical patients in this age group while excluding clinically irrelevant cases.
Recruitment	5	Very pragmatic: Patients are seen routinely in the outpatient clinic and are recruited from this usual care pathway.
Setting	5	Very pragmatic: Conducted at two hospital centers in Denmark and Finland within standard clinical settings, without any trial-specific environments
Organisation	5	Very pragmatic: Standard surgical and non-surgical care is delivered by usual clinical staff, with physiotherapy provided in municipalities. Interventions are delivered according to routine practice.
Flexibility (delivery)	5	Very pragmatic: Surgical and non-surgical interventions are delivered according to standard protocols with flexibility, reflecting usual care (e.g., choice of plate or nail, optional 12-week visit in the non-surgical group).
Flexibility (adherence)	4	Rather pragmatic: The intervention itself (surgical or non-surgical treatment) does not require active patient adherence, reflecting usual care. Patients are only required to participate in post-intervention rehabilitation, which is delivered according to usual care with individualized duration, intensity, and exercises in collaboration with a physiotherapist. Adherence is defined as completion of a minimum of 6 weeks of rehabilitation
Follow-up	4	Rather pragmatic: Follow-up assessments are mostly aligned with routine care; however, it is not usual care to follow up all patients at 6 months with PROMs (OSS, EQ-5D). Extensive data are collected, including all complications and weeks of rehabilitation. PROMs are collected via REDCap, with minimal additional burden during the 6-month follow-up visit
Primary outcome	5	Very pragmatic: The 12-month OSS is patient-reported and captures pain, function, sleep, and quality of life, making it clearly important to participants. Assessment is pragmatic and minimally intrusive
Primary analysis	5	Very pragmatic: Intention-to-treat analysis, linear mixed models for repeated measures; pragmatic in analyzing outcomes as they occur in clinical practice.
Sum total	42	Pragmatic trial.<br>The average PRECIS-2 score is 4.67.<br>Because the average exceeds 3 points, it can be classified as a pragmatic trial [20].

Supplementary information, file 1:
Statistical analysis plan (SAP)

Administrative information

Statistical analysis plan for the study" Surgical versus non-surgical treatment of displaced proximal humerus fracture in adults aged 50 to 65 years: a protocol for a pragmatic randomized controlled trial".

Trial registration number: Clinicaltrials.gov, NCT06416618

SAP version 1, date: 12th September 2025

This document has been written based on information contained in the study protocol version version: 1

The statistical analysis will follow the Guidelines for the Content of Statistical Analysis Plans in Clinical Trials [22]

SAP revision history

Protocol version	Updated SAP version no.	Section number changed	Description of and reason for change	Date changed	Timing in relation to interim analyses.
1	NA	NA	NA	NA	NA

Roles and responsibilities:

Line Houkjær (L.H), chief investigator/clinical lead, writing of SAP, responsible for statistical analysis with supervision from biostatistican from University of Copenhagen. Affiliations: Centre for Evidence-Based Orthopaedics, Zealand University Hospital, Køge.

Stig Brorson (S.B), supervisor, Centre for Evidence-Based Orthopaedics, Zealand University Hospital, Køge and Department of Clinical Medicine, University of Copenhagen

Antti P. Launonen (A.L), co-supervisor, Faculty of Medicine and Health Technology, Tampere University Hospital, Finland

Introduction

Meta-analyses comparing operative and non-operative treatments for PHF have reached discordant conclusions and suffer from critically low methodological quality [6]. A Cochrane review reported high-certainty evidence of no clinically important difference between surgical and non-surgical treatment in patient-reported shoulder function one and two years after injury [7]. Most included studies focus on older patients; however, a subgroup analysis from the ProFHER study [8] of adults aged 16–65 found no significant differences in patient-reported shoulder function (Oxford Shoulder Score (OSS)) between surgical and non-surgical treatment at 6 months, one year and two years, with overlapping confidence intervals. There was a trend toward more rapid improvement among younger patients (< 65 years) in the non-surgical group compared to those in the surgical group.

The treatment of choice for displaced PHF in adults 50-65 years is anatomical reconstruction, and osteosynthesis if possible, which is why many patients in this age group with a displaced PHF undergo surgical treatment. There exists an intermediate age group of patients for whom it remains uncertain whether the benefits and harms of non-surgical and surgical interventions are comparable.

This study aims to evaluate whether osteosynthesis is superior to non-surgical treatment measured by patient-reported shoulder function after 12-month in patients aged 50 to 65 years with displaced proximal humerus fractures.

Hypothesis

The null hypothesis is that the shoulder function measured with Oxford Shoulder Score 12 months after surgery is not superior to non-surgical treatment.

The alternative hypothesis is that the shoulder function measured with Oxford Shoulder Score 12 months after surgery is superior to non-surgical treatment.

Study Method

Trial design

The study is a two-center, parallel-group, randomized, superiority trial comparing osteosynthesis with non-surgical treatment following a displaced PHF in patients aged 50-65 years with a 1:1 allocation ratio. Patients are randomized to either surgical or non-surgical treatment.

Randomization

The randomization process is described in full within the clinical trial protocol.
All investigators will be blinded to the allocation table. We will be using block randomization, including location and gender stratification (shifting block sizes of 4 and 6 in each block).

Sample size and power consideration

The standard deviation has been reported as 12 for OSS [34]. The Minimal Clinically Important Difference (MCID) for the OSS (0-48) has not been established [35]. Without a patient-derived MCID for PHF, a relevant difference of 9,6 was assumed to represent clinically meaningful difference. This is equivalent to approximately a 20 % difference between the surgical group and the non-surgical group on a 0–48 OSS scale. With a power of 80% and a 5% level of significance, a sample size of 25 participants per group is required. Assuming a 15 % loss to follow-up, the recruitment target is 30 patients in each group, meaning 60 participants overall. The recruitment period is expected to be 24 months.

Interim analysis

We plan to conduct an interim analysis when 50% (n = 30) of the included patients have responded to the OSS after 6 months. The results from the interim analysis will not be published separately. It will be part of the final article.

The significance level for the interim analysis will be adjusted using the O'Brien-Fleming method. For the interim analysis, the significance level will be set at 0.001. The final analysis, after all participants have been included, will have a significance level of 0.049.

By a third person, the data will be blinded to the treatment location before the interim analysis is conducted. A data management committee will be organized to monitor and evaluate the data. Intervention-related hospitalization or mortality, while unexpected, will be reported to the ethics committee.

The data management committee will monitor unexpected unwanted events, continued inclusion will be considered if the failure rate is above 30 %. Regardless of whether the interim analyses suggest that the study is unlikely to achieve its primary objectives, the study will proceed as initially planned.

Trial Population

Inclusion and exclusion criteria are available in protocol.

Patients eligible for inclusion in the trial are those aged between 50 and 65 years who have sustained a displaced proximal humerus fracture following a low-energy trauma. In addition, patients must be cognitively capable of understanding and completing follow-up questionnaires.

Patients will be excluded from the trial if they are unable to understand written and spoken language in Danish or Finnish/Swedish, or if they are unable to provide informed consent. Fracture-related exclusion criteria include fractures assessed as non-reconstructable by the treating surgeon; isolated tuberosity fractures, fracture dislocations, open fractures, or fractures involving the articular surface; fractures with less than 25% contact between the head fragment and the metaphysis/diaphysis, measured on two perpendicular radiographs taken two weeks post-injury; pathological fractures; previous fractures in the same proximal humerus; and concomitant fractures that may influence the outcome. Patients with paralysis of the affected upper extremity will also be excluded.

A CONSORT flow diagram (Figure 1) will be used to summarize:

<u>Screening data</u>

The number of patients screened, the number of patients recruited, the number of screened patients not recruited, and the reason for non-recruitment. This summary will be provided overall and by study centre.

<u>Recruitment</u>

- assessed for eligibility at screening
  - eligible at screening
  - ineligible at screening*
- eligible and randomised
- eligible but not randomised*
- received the randomised allocation
- did not receive the randomised allocation*

- lost to follow-up*
- discontinued the intervention*
- randomised and included in the primary analysis
- randomised and excluded from the primary analysis*

*reasons will be provided.

Decliner cohort

Eligible patients who choose not to participate will be recorded, and, if possible, their reasons will be noted. Individuals who decline to participate will be asked if they are willing to remain part of the study as members of the decliner cohort. The decliner cohort will receive the same treatment, follow-up, and outcome measurements as the included patients.

[Tabel 1]

Baseline patient characteristics

Baseline characteristics will be presented using descriptive statistics. We will compare the following patient characteristics between the groups at baseline: age (categorized into 5-year age groups), sex, height, weight, ASA score, fracture classification (Neer [5]), dominant arm (yes/no), smoking, alcohol consumption, educational level, working status, work type, and clinical frailty scale. SD will be reported, and n (%) for categorical values.

Analysis

The schedule of study and expected visit dates are defined in Figure 2 in the protocol.

Outcome definition

The primary outcome will be the Oxford Should Score (OSS) at 12-months. The secondary outcome will be OSS at 6 and 24 months and EQ-5D-3L score measured at the same time-points. Adverse events will be registered at follow-ups. Conversion to surgery will be registered. OSS and EQ-5D-3L will be completed just before the 6 months visit. At 12 and 24 months, they will be completed by mail. DXA scan is planned to be done within first 6 months from injury.

Oxford Sholder Score is a patient-administrated questionnaire to assess shoulder function. It consists of 12 questions on a 5-point Likert scale (both pain- and function-related), each offering five ordinal response options. The cumulative score ranges between 0 and 48, being 0 (worst) to 48 (best: no pain or functional limitation).

EQ-5D-3L is a generic health-related quality-of-life assessment tool. It consists of a 5-dimension descriptive questionnaire [28]. Each dimension has 3 levels: No problems, some/moderate problems and unable/extreme problems. Each dimension is weighted differently and results in a total score based on the EQ-5D index calculator (TTO). The EQ-5D-3L also includes an overall scale (numeric rating scale) in which patient rates overall health condition today on a scale between 1 - 100, with 100 being the highest level of health imaginable

Blinding

The primary investigator will conduct the blinded statistical analysis through data anonymization and will be supervised by a biostatistician assigned by the University of Copenhagen. After the blinded statistical analysis has been completed, two abstracts will be written based on the pre-blinding analysis results before the blinding is revealed.

Data management

B.O. and AL will have access to data from Finland, L.H. will have access to all data. An information security committee has approved the server at Region Zealand University Hospital. Data handling and processing procedures will be conducted in accordance with the approved Data Management Plan for the trial, and is available upon request.

Analysis method

In all the analysis 95% confidence intervals will be calculated, and statistical significance will be assessed using p-values.
Final analysis will be conducted, when outcome from 6, 12 and 24 months are available from all patients.

The OSS results for surgical and non-surgical treatment groups at 6, 12, and 24 months will be tabulated and graphically represented with mean, SD, median, and IQR according to the distribution.

The primary outcome, OSS at 12 months, will be analysed using two-sample t-test comparing the two treatment groups. Normality assumptions will be assessed, and if violated, alternative methods such as transformation or non-parametric Mann–Whitney U test will be considered. As suggested in the extension of the CONSORT 2010 statement, the primary outcome will be analysed for the intention-to-treat (ITT) population.

In addition, linear mixed models will be used to analyse OSS and EQ-5D over time (6, 12 and 24 months) accounting for the repeated measures structure and to explore outcome trajectories. Model-based contrasts from the LMM will be used to estimate adjusted between-group differences at specific time points. Missing data will be handled within the mixed model framework, assuming data are missing at random

Osteoporosis will be collected and reported descriptively as a three-level ordinal variable based on T-scores (osteoporosis, osteopenia, and normal bone density).

Handling of missing data

Electronic responses are submitted via a link, ensuring no missing data using 'required fields' in REDCap.

We will distinguish between individual item missing (one answer missing), item-wise missing (more than one answer in questionnaire missing) and case-wise missing (all answer in questionnaire missing).

Patient-reported outcome data will be entered directly into the REDCap system by the patients using the 'required fields' option activated to help ensure limited missing items from the completed questionnaires. If missing individual items in OSS and EQ-5D-3L should occur, we will contact the participant. If not possible, the item will be considered missing at random and will be substituted by the average value of other items. Missing more than one question, will be handles as case-wise missing. In case-wise of missing data, the linear mixed model can handle data points within the model, assuming dropout is missing at random.

Additional analysis

Adverse events

We will record and report adverse events in each group.

Rehabilitation

At the 6-month check-up patients will be asked, and we will record and report the length of rehabilitation in municipalities (in weeks) and whether the patient has followed the rehabilitation plan.

Salvage procedure

Salvage procedures and adverse events will be reported descriptively. Reoperation in the

form of hardware removal will be classified as a complication, not a salvage procedure. There will be two analyses for ITT: one including salvage procedures and one excluding them. The timing of salvage procedures will be analyzed using a Cox proportional hazards model to compare time to salvage between treatment groups.

Decliner cohort:
Patients who declined randomization but consented to follow-up will form the decliner cohort. Baseline characteristics will be summarized descriptively and compared between the randomized and decliner cohorts, and between decliners by treatment choice. The primary outcome (OSS at 12 months) will be analyzed by treatment modality. Secondary outcome measures (OSS and EQ-5D at 6, 12, and 24 months) will be presented descriptively and graphically. Analyses will include multivariable linear regression adjusted for baseline covariates; propensity score methods may be applied. The incidence and timing of salvage procedures will be described and analyzed using Cox proportional hazards models.

Harms

Serious adverse events will be reported to a data management committee. Other adverse effects will be recorded and reported in event groups, which are elucidated in Appendix 2. All adverse effects will be reported descriptively.

Statistical software

Data will be analysed using the statistical "R" [37].

Withdrawal

The level of withdrawal will be monitored and reported separately for withdrawal from the intervention and from follow-up. Withdrawal from the intervention is defined as patients who discontinue or decline the allocated treatment after randomisation but remain in the trial for follow-up. Withdrawal from follow-up is defined as patients who actively withdraw consent for continued data collection or are lost to follow-up despite repeated contact attempts. The extent and timing of withdrawal will be summarised descriptively for each trial arm. Reasons for withdrawal, if known, will be documented.

Adherence and protocol violations

Patients who drop out of the trial will be noted along with treatment group and reasons. Numbers and time for loss to follow up will be reported descriptively.

Non statistical methods

No nonstandard statistical methods is planned. If any are applied during the analysis in the final report, appropriate references will be provided to justify their use.

Trial Master File and Statistical Master File*

All documentation related to trial conduct and statistical analyses will be stored in the Trial Master File (TMF) and Statistical Master File (SMF), respectively.

Supplementary information, file 3:

Checklists

SPIRIT CONSORT and SAP Checklist

SPIRIT Checklist for *Trials*

Complete this checklist by entering the page and line numbers where each of the items listed below can be found in your manuscript.

Your manuscript may not currently address all the items on the checklist. Please modify your text to include the missing information. If you are certain that an item does not apply, please state "n/a" and provide a short explanation. **Leaving an item blank or stating "n/a" without an explanation will lead to your manuscript being returned before review.**

Upload your completed checklist as an additional file when you submit to *Trials*. You must reference this additional file in the main text of your protocol submission. The completed SPIRIT figure must be included within the main body of the protocol text and can be downloaded here: http://www.spirit-statement.org/schedule-of-enrolment-interventions-and-assessments/

In your methods section, please state that you used the SPIRIT reporting guidelines, and cite them as:

Chan A-W, Tetzlaff JM, Gøtzsche PC, Altman DG, Mann H, Berlin J, Dickersin K, Hróbjartsson A, Schulz KF, Parulekar WR, Krleža-Jerić K, Laupacis A, Moher D. SPIRIT 2013 Explanation and Elaboration: Guidance for protocols of clinical trials. BMJ. 2013;346:e7586

		Reporting Item	Page and Line Number	Reason if not applicable
**Administrative information**				
Title	#1	Descriptive title identifying the study design, population, interventions, and, if applicable, trial acronym	Page 1<br>Line 3-5	
Trial registration	#2a	Trial identifier and registry name. If not yet registered, name of intended registry	Page 1<br>Line 6-8	
Trial registration: data set	#2b	All items from the World Health Organization Trial Registration Data Set	Page 1<br>Line 6-8	
Protocol version	#3	Date and version identifier	Page1<br>Line 9-10	

Funding	#4	Sources and types of financial, material, and other support	Page 16-17<br>Line 23-6	
Roles and responsibilities: contributorship	#5a	Names, affiliations, and roles of protocol contributors	Page 1<br>Line 14-27	
Roles and responsibilities: sponsor contact information	#5b	Name and contact information for the trial sponsor	Page 2<br>Line 1-2	
Roles and responsibilities: sponsor and funder	#5c	Role of study sponsor and funders, if any, in study design; collection, management, analysis, and interpretation of data; writing of the report; and the decision to submit the report for publication, including whether they will have ultimate authority over any of these activities	Page 2<br>Line 3-6	
Roles and responsibilities: committees	#5d	Composition, roles, and responsibilities of the coordinating centre, steering committee, endpoint adjudication committee, data management team, and other individuals or groups overseeing the trial, if applicable (see Item 21a for data monitoring committee)	Page 13<br>line 24-29 and page 17<br>line 18-19	
**Introduction**				
Background and rationale	#6a	Description of research question and justification for undertaking the trial, including summary of relevant studies (published and unpublished) examining benefits and harms for each intervention	Page 5-6<br>Line 1-10	

Background and rationale: choice of comparators	#6b	Explanation for choice of comparators	Page 5-6<br>Line 11-7	
Objectives	#7	Specific objectives or hypotheses	Page 6, Line 8-10<br><br>Page 11, line 13-17	
Trial design	#8	Description of trial design including type of trial (eg, parallel group, crossover, factorial, single group), allocation ratio, and framework (eg, superiority, equivalence, non-inferiority, exploratory)	Page 6<br>Line 13-16	
**Methods: Participants, interventions, and outcomes**				
Study setting	#9	Description of study settings (eg, community clinic, academic hospital) and list of countries where data will be collected. Reference to where list of study sites can be obtained	Page 6<br>Line 25-26	
Eligibility criteria	#10	Inclusion and exclusion criteria for participants. If applicable, eligibility criteria for study centres and individuals who will perform the interventions (eg, surgeons, psychotherapists)	Page 6-7<br>Line 27-15	
Interventions: description	#11a	Interventions for each group with sufficient detail to allow replication, including how and when they will be administered	Page 8-9<br>Line 12-11	
Interventions: modifications	#11b	Criteria for discontinuing or modifying allocated interventions for a given trial participant (eg, drug	Page 9<br>Line 12-18	

		dose change in response to harms, participant request, or improving / worsening disease)		
Interventions: adherance	#11c	Strategies to improve adherence to intervention protocols, and any procedures for monitoring adherence (eg, drug tablet return; laboratory tests)	Page 9<br>line 19-29	
Interventions: concomitant care	#11d	Relevant concomitant care and interventions that are permitted or prohibited during the trial	Page 9<br>Line 6-11	
Outcomes	#12	Primary, secondary, and other outcomes, including the specific measurement variable (eg, systolic blood pressure), analysis metric (eg, change from baseline, final value, time to event), method of aggregation (eg, median, proportion), and time point for each outcome. Explanation of the clinical relevance of chosen efficacy and harm outcomes is strongly recommended	Page 10-11<br>line 1-11	
Participant timeline	#13	Time schedule of enrolment, interventions (including any run-ins and washouts), assessments, and visits for participants. A schematic diagram is highly recommended (see Figure)	Figure 1 and 2	
Sample size	#14	Estimated number of participants needed to achieve study objectives and how it was determined, including clinical and statistical assumptions supporting any sample size calculations	Page 11<br>line 19-27	

Recruitment	#15	Strategies for achieving adequate participant enrolment to reach target sample size	Page 7<br>line 16-24	
**Methods: Assignment of interventions (for controlled trials)**				
Allocation: sequence generation	#16a	Method of generating the allocation sequence (eg, computer-generated random numbers), and list of any factors for stratification. To reduce predictability of a random sequence, details of any planned restriction (eg, blocking) should be provided in a separate document that is unavailable to those who enrol participants or assign interventions	Page 8<br>line 3-9	
Allocation concealment mechanism	#16b	Mechanism of implementing the allocation sequence (eg, central telephone; sequentially numbered, opaque, sealed envelopes), describing any steps to conceal the sequence until interventions are assigned	Page 8<br>line 3-11	
Allocation: implementation	#16c	Who will generate the allocation sequence, who will enrol participants, and who will assign participants to interventions	Page 8<br>line 3-11	
Blinding (masking)	#17a	Who will be blinded after assignment to interventions (eg, trial participants, care providers, outcome assessors, data analysts), and how	Page 8, line 6 and page 13, line 10-17	
Blinding (masking): emergency unblinding	#17b	If blinded, circumstances under which unblinding is permissible, and procedure for revealing a		No unblinding of allocation sequence or data will be permissible.

		participant's allocated intervention during the trial		
**Methods: Data collection, management, and analysis**				
Data collection plan	#18a	Plans for assessment and collection of outcome, baseline, and other trial data, including any related processes to promote data quality (eg, duplicate measurements, training of assessors) and a description of study instruments (eg, questionnaires, laboratory tests) along with their reliability and validity, if known. Reference to where data collection forms can be found, if not in the protocol	Page 8, line 12-30<br>Page 9, line 1-29<br>Page 10-11, line 6-5	
Data collection plan: retention	#18b	Plans to promote participant retention and complete follow-up, including list of any outcome data to be collected for participants who discontinue or deviate from intervention protocols	Page 9, line 19-29<br>Page 14, line 7-10	
Data management	#19	Plans for data entry, coding, security, and storage, including any related processes to promote data quality (eg, double data entry; range checks for data values). Reference to where details of data management procedures can be found, if not in the protocol	Page 14, line 1-6	
Statistics: outcomes	#20a	Statistical methods for analysing primary and secondary outcomes. Reference to where other details of the statistical analysis plan can be found, if not in the protocol	Page 11, line 29 to page 12, line 21	

Statistics: additional analyses	#20b	Methods for any additional analyses (eg, subgroup and adjusted analyses)	Page 12, line 22 to page 14, line 9	
Statistics: analysis population and missing data	#20c	Definition of analysis population relating to protocol non-adherence (eg, as randomised analysis), and any statistical methods to handle missing data (eg, multiple imputation)	Page 12 line 20-21. Details are provided in Supplementary Information, file 4.	
**Methods: Monitoring**				
Data monitoring: formal committee	#21a	Composition of data monitoring committee (DMC); summary of its role and reporting structure; statement of whether it is independent from the sponsor and competing interests; and reference to where further details about its charter can be found, if not in the protocol. Alternatively, an explanation of why a DMC is not needed	Page 13 line 24-29<br><br>And page 15, line 18-19	
Data monitoring: interim analysis	#21b	Description of any interim analyses and stopping guidelines, including who will have access to these interim results and make the final decision to terminate the trial	Page 13, line 18-29	
Harms	#22	Plans for collecting, assessing, reporting, and managing solicited and spontaneously reported adverse events and other unintended effects of trial interventions or trial conduct	Page 10, line 24-28	
Auditing	#23	Frequency and procedures for auditing trial conduct, if any, and whether the process will be independent from investigators and the sponsor		No formal independent auditing is planned. The trial may be subject to

				inspection by the relevant regulatory authorities or ethics committees
**Ethics and dissemination**				
Research ethics approval	#24	Plans for seeking research ethics committee / institutional review board (REC / IRB) approval	Page 15 line 27 to page 16 line 2	
Protocol amendments	#25	Plans for communicating important protocol modifications (eg, changes to eligibility criteria, outcomes, analyses) to relevant parties (eg, investigators, REC / IRBs, trial participants, trial registries, journals, regulators)	Page 16 line 6-9.	
Consent or assent	#26a	Who will obtain informed consent or assent from potential trial participants or authorised surrogates, and how (see Item 32)	Page 7 line 23-24	
Consent or assent: ancillary studies	#26b	Additional consent provisions for collection and use of participant data and biological specimens in ancillary studies, if applicable		N/A No biological specimens were collected as part of this trial
Confidentiality	#27	How personal information about potential and enrolled participants will be collected, shared, and maintained in order to protect confidentiality before, during, and after the trial	Page 9 line 23-24	
Declaration of interests	#28	Financial and other competing interests for principal investigators for the overall trial and each study site	Page 16, line 24-25	
Data access	#29	Statement of who will have access to the final trial dataset, and disclosure of contractual	Page 17 line 10-17	

		agreements that limit such access for investigators		
Ancillary and post trial care	#30	Provisions, if any, for ancillary and post-trial care, and for compensation to those who suffer harm from trial participation		N/A No provision
Dissemination policy: trial results	#31a	Plans for investigators and sponsor to communicate trial results to participants, healthcare professionals, the public, and other relevant groups (eg, via publication, reporting in results databases, or other data sharing arrangements), including any publication restrictions	Page 16, line 23	
Dissemination policy: authorship	#31b	Authorship eligibility guidelines and any intended use of professional writers	Page 17 line 10-17	
Dissemination policy: reproducible research	#31c	Plans, if any, for granting public access to the full protocol, participant-level dataset, and statistical code		No plans
**Appendices**				
Informed consent materials	#32	Model consent form and other related documentation given to participants and authorised surrogates		Model informed consent materials are available from the corresponding author on reasonable request
Biological specimens	#33	Plans for collection, laboratory evaluation, and storage of biological specimens for genetic or molecular analysis in the current trial and for future use in ancillary studies, if applicable		N/A No biological specimens were collected as part of this trial

It is strongly recommended that this checklist be read in conjunction with the SPIRIT 2013 Explanation & Elaboration for important clarification on the items. Amendments to the protocol should be tracked and dated. The SPIRIT checklist is copyrighted by the SPIRIT Group under the Creative Commons "Attribution-NonCommercial-NoDerivs 3.0 Unported" license. This checklist can be completed online using https://www.goodreports.org/, a tool made by the EQUATOR Network in collaboration with Penelope.ai

Statistical Analysis Plan (SAP) Checklist v 1.0 2019

Section/Item	Index	Description	Reported on page #
**Section 1: Administrative information**			
Trial and Trial registration	1a	Descriptive title that matches the protocol, with SAP either as a forerunner or subtitle, and trial acronym (if applicable)	Supl. file 1 p. 1
	1b	Trial registration number	Supl. file 1 p. 1
SAP Version	2	SAP version number with dates	Supl. file 1 p. 1
Protocol Version	3	Reference to version of protocol being used	Supl. file 1 p. 1
SAP revisions	4a	SAP revision history	Supl. file 1 p. 1
	4b	Justification for each SAP revision	Supl. file 1 p. 1
	4c	Timing of SAP revisions in relation to interim analyses, etc.	Supl. file 1 p. 1
Roles and responsibility	5	Names, affiliations, and roles of SAP contributors	Supl. file 1 p. 1
Signatures of:	6a	Person writing the SAP	Supl. file 1 p. 1
	6b	Senior statistician responsible	Supl. file 1 p. 1
	6c	Chief investigator/clinical lead	Supl. file 1 p. 1
**Section 2: Introduction**			
Background and rationale	7	Synopsis of trial background and rationale including a brief description of research question and brief justification for undertaking the trial	Supl. file 1 p. 3
Objectives	8	Description of specific objectives or hypotheses	Supl. file 1 p. 3
**Section 3: Study Methods**			
Trial design	9	Brief description of trial design including type of trial (e.g., parallel group, multi-arm, crossover, factorial)<br>and allocation ratio and may include brief description of interventions	Supl. file 1 p. 4
Randomization	10	Randomization details, e.g., whether any minimization or stratification occurred (including stratifying<br>factors used or the location of that information if it is not held within the SAP)	Supl. file 1 p. 4
Sample size	11	Full sample size calculation or reference to sample size calculation in protocol (instead of replication in SAP)	Supl. file 1 p. 4

Framework	12	Superiority, equivalence, or noninferiority hypothesis testing framework, including which comparisons will be presented on this basis	Supl. file 1 p. 4
Statistical interim analysis and stopping guidance	13a	Information on interim analyses specifying what interim analyses will be carried out and listing of time points	Supl. file 1 p. 4
	13b	Any planned adjustment of the significance level due to interim analysis	Supl. file 1 p. 4
	13c	Details of guidelines for stopping the trial early	Supl. file 1 p. 5
Timing of final analysis	14	Timing of final analysis, e.g., all outcomes analysed collectively or timing stratified by planned length of follow-up	Supl. file 1 p. 6
Timing of outcome assessments	15	Time points at which the outcomes are measured including visit “windows”	Supl. file 1 p. 6
**Section 4: Statistical Principals**			
Confidence intervals and *P* values	16	Level of statistical significance	Supl. file 1 p. 4
	17	Description and rationale for any adjustment for multiplicity and, if so, detailing how the type 1 error is to be controlled	Supl. file 1 p. 4
	18	Confidence intervals to be reported	Supl. file 1 p. 7
Adherence and Protocol deviations	19a	Definition of adherence to the intervention and how this is assessed including extent of exposure	Supl. file 1 p. 9
	19b	Description of how adherence to the intervention will be presented	Supl. file 1 p. 9
	19c	Definition of protocol deviations for the trial	Supl. file 1 p. 9
	19d	Description of which protocol deviations will be summarized	Supl. file 1 p. 9
Analysis populations	20	Definition of analysis populations, e.g., intention to treat, per protocol, complete case, safety	Supl. file 1 p. 9
**Section 5: Trial Population**			
Screening data	21	Reporting of screening data (if collected) to describe representativeness of trial sample	Supl. file 1 p. 5
Eligibility	22	Summary of eligibility criteria	Supl. file 1 p. 6
Recruitment	23	Information to be included in the CONSORT flow diagram	Supl. file 1 p. 6
Withdrawal/ Follow-up	24a	Level of withdrawal, e.g., from intervention and/or from follow-up	Supl. file 1 p. 9
	24b	Timing of withdrawal/lost to follow-up data	Supl. file 1 p. 9
	24c	Reasons and details of how withdrawal/lost to follow-up data will be presented	Supl. file 1 p. 9

Baseline patient characteristics	25a	List of baseline characteristics to be summarized	Supl. file 1 p. 6
	25b	Details of how baseline characteristics will be descriptively summarized	Supl. file 1 p. 6
**Section 6: Analysis**			
Outcome definitions		List and describe each primary and secondary outcome including details of:	Supl. file 1 p. 6
	26a	Specification of outcomes and timings. If applicable include the order of importance of primary or key secondary end points (e.g., order in which they will be tested)	Supl. file 1 p. 6
	26b	Specific measurement and units (e.g., glucose control, hbA1c [mmol/mol or %])	Supl. file 1 p. 6+7 (OSS + EQ-5D)
	26c	Any calculation or transformation used to derive the outcome (e.g., change from baseline, QoL score, Time to event, logarithm, etc.)	None
Analysis methods	27a	What analysis method will be used and how the treatment effects will be presented	Supl. file 1 p. 6
	27b	Any adjustment for covariates	None
	27c	Methods used for assumptions to be checked for statistical methods	Supl. file 1 p. 7
	27d	Details of alternative methods to be used if distributional assumptions do not hold, e.g., normality,proportional hazards, etc.	Supl. file 1 p. 7
	27e	Any planned sensitivity analyses for each outcome where applicable	Supl. file 1 p. 7
	27f	Any planned subgroup analyses for each outcome including how subgroups are defined	No subgroup analyis
Missing data	28	Reporting and assumptions/statistical methods to handle missing data (e.g., multiple imputation)	Supl. file 1 p. 8
Additional analyses	29	Details of any additional statistical analyses required, e.g., complier-average causal effect10 analysis	None
Harms	30	Sufficient detail on summarizing safety data, e.g., information on severity, expectedness, and causality; details of how adverse events are coded or categorized; how adverse event data will be analysed, i.e., grade 3/4 only, incidence case analysis, intervention emergent analysis	Supl. file 1 p. 8
Statistical software	31	Details of statistical packages to be used to carry out analyses	Supl. file 1 p. 9
References	32a	References to be provided for nonstandard statistical methods	Supl. file 1 p. 10
	32b	Reference to Data Management Plan	Supl. file 1 p. 7
	32c	Reference to the Trial Master File and Statistical Master File	Supl. file 1 p. 10

	32d	Reference to other standard operating procedures or documents to be adhered to	None other

Taken from the paper: Gamble C, Krishan A, Stocken D, Lewis S, Juszczak E, Doré C, et al. Guidelines for the Content of Statistical Analysis Plans in Clinical Trials. JAMA. 2017;318(23):2337-43.

Abbreviations: CONSORT, Consolidated Standards of Reporting Trials; hbA1c, haemoglobin A1c; QoL, quality of life; SAP, statistical analysis plan.

For more information visit:

The development of this checklist was funded by the MRC Hubs for Trials Methodology Research

Supplementary information, File 4
Rehabilitation: Patient information sheet

Operative group

The weeks count from the surgery date.

Week 0-6:
We encourage daily exercises for free movement of the hand, wrist, elbow, and neck in their full range of motion.

We recommend using the arm for daily activities as soon as the pain allows. You can perform free movement exercises; however, avoid outward rotation beyond the neutral position (pointing forward) and any inward rotation as if putting the hand behind the back. Include passive movements to enhance mobility, gradually shifting the focus towards more active movements.

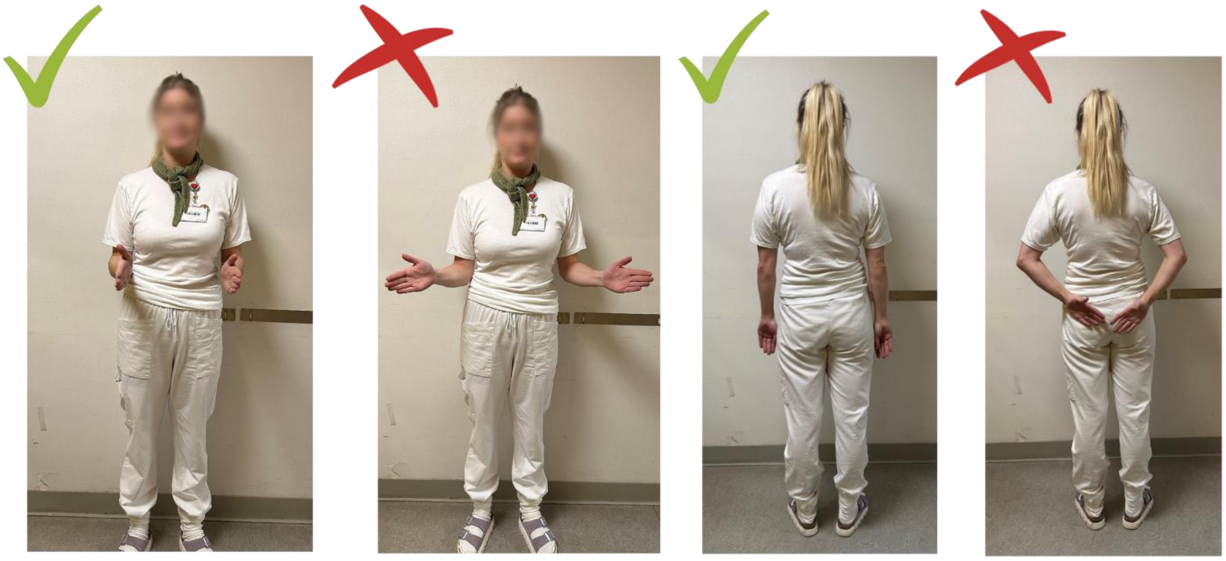

Week 6-12:
Now you can engage in exercises with free movement and gradually increasing the load.

From week 12:
Now, full load is allowed.

Non-surgical group

The weeks count from the injury date.

Week 0-6:
We encourage daily exercises for free movement of the hand, wrist, elbow, and neck in their full range of motion.

We recommend using the arm for daily activities as soon as the pain allows. You can perform free movement exercises; however, avoid outward rotation beyond the neutral position (pointing forward) and any inward rotation as if putting the hand behind the back. Include passive movements to enhance mobility, gradually shifting the focus towards more active movements.

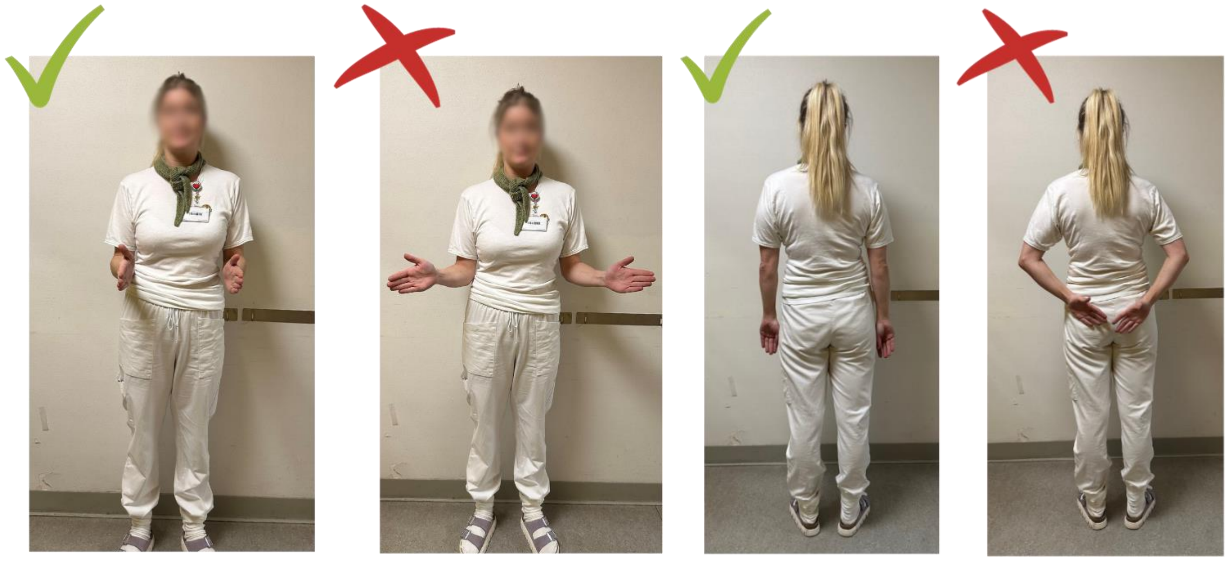

Week 6-12:
Now you can engage in exercises with free movement and gradually increasing the load.

From week 12:
Now, full load is allowed.

Supplementary information, file 5: Complications

**Event group**	**Specification**	**Surgical**	**Non-surgical**	**Secondary RSA**
**Implant (device) (postoperative)**	Distinction made between:			
	• Malposition	x		x
	• Screw back out	x		
	• Implant breakage	x		x
	• Migration	x		x
	• Implant loosening	x		x
**Osteochondral**	Distinction made between:			
	• New fracture: around the implant	x		
	• Screw cutout	x		
	• Loss of fracture reduction	x		
	• Head necrosis	x	x	
	• Tuberosity migration / resorption	x	x	
	• Other bone formation/resorption	x	x	
	• Delayed union / nonunion	x	x	
**Shoulder instability**	Symptomatic shoulder instability			x
**Peripheral neurological**	Sensory and/or motor disturbance<br>Complex regional pain syndrome (CRPS)	x	x	x
**Vascular**	Events injury to an artery or vein at the injured arm, such as ischemia, thrombosis or hematoma (requiring intervention)	x	x	x
**Infections**	Sinus Tract or Synovial WCC >3000 (PMN% >80%) or 2 positive samples with the same microorganism [45]	x		x
**Device**	Events used to immobilize the arm to support the fracture, e.g. local reactions such as skin lesions	x	x	x
**Superficial soft tissue**	Events affecting the superficial soft tissues at and around the surgical site, that require additional treatment, e.g, delayed wound healing; hypersensitivity reaction; skin necrosis; skin bulla, hypertrophic scar and keloid	x		x
**Deep soft tissue**	- External muscular envelope: deltoid-pectoralis major<br>- Subacromial pain<br>- Rotator cuff muscle-tendon and biceps tendon	x	x	x
**Other complications**	Persistent severe pain	x	x	x

Supplementary Information, File 6

Ethical approval

In Denmark and Finland

Line Houkjær
Sjællands Universitets Hospital, Køge
Ortopædkirurgisk afdeling
Lykkebækvej 1
4600 Køge

Dato: 6. maj 2024

Sags ID: EMN-2024-01516
Dokument ID: 11448746

Den Videnskabsetisk Komite for Region Sjælland
Lykkebækvej 1
4600 Køge

Tlf.: 30891309
www.regionsjaelland.dk

E-mail: rvk-sjaelland@regionsjaelland.dk

Endelig godkendelse af forsøg SJ-1063, "Forsøgets titel (engelsk): Surgical versus non-surgical treatment of displaced proximal humerus fracture in adults aged 50 to 65 years: a pragmatic randomized controlled trial."

Forsøgets titel (dansk): "Kirurgisk eller ikke-kirurgisk behandling af forskudte brud på den øverste del af overarmen i aldersgruppen 50-65 år."

Afgørelsen er truffet efter lovbekendtgørelse nr. 1338 af 1. september 2020 om videnskabsetisk behandling af sundhedsvidenskabelige forskningsprojekter og sundhedsdatavidenskabelige forskningsprojekter (komitéloven).

Den Videnskabsetiske Komité for Region Sjælland bekræfter modtagelsen af mail af 2. april 2024, som svar på komiteens afgørelse af 14. marts 2024, hvori der opstilledes betingelser for godkendelsen af projektet.

Betingelserne for godkendelsen anses for opfyldt. Projektet er dermed endeligt godkendt.

Godkendelsen gælder til den 1. februar 2027 og omfatter følgende dokumenter:

- Forsøgsprotokol, version 2, af 18. februar 2024 med de til protokollen tilhørende spørgeskemaer,
- Deltagerinformation, version 2 af 18. marts 2024
- Informeret samtykkeerklæring, version 1, af 2. februar 2024

Godkendelsen gælder for de anmeldte forsøgssteder og den anmeldte forsøgsansvarlige i Danmark.

Det bemærkes, at komiteen ikke er ressortmyndighed for regelsættet om databeskyttelse. Komiteen forudsætter at projektet gennemføres og

Region Sjælland anvender de personoplysninger, du giver os i forbindelse med din henvendelse. Du kan læse mere om formålet med anvendelsen samt dine rettigheder på vores hjemmeside nederst på regionens hjemmeside.

informationen til deltagerne gives i overensstemmelse databeskyttelsesforordningen og databeskyttelsesloven.

Vær opmærksom på, at den dataansvarlige efter databeskyttelsesforordningen har pligt til at underrette de registrerede om, at der sker behandling af oplysninger. Databeskyttelsesforordningen indeholder en række krav til bl.a. indhold og form af underretningen. Læs mere om oplysningspligten (art. 13 og 14) og om de registreredes øvrige rettigheder i Datatilsynets vejledning om de registreredes rettigheder (se www.datatilsynet.dk).

Iværksættelse af projektet i strid med godkendelsen kan straffes med bøde eller fængsel, jf. komitélovens § 41.

Ændringer

Foretages der væsentlige ændringer i protokolmaterialet under gennemførelsen af projektet, skal disse anmeldes til komiteen i form af tillægsprotokoller. Ændringerne må først iværksættes efter godkendelse fra Komiteen, jf. komitélovens § 27, stk. 1.

Anmeldelse af tillægsprotokoller skal ske elektronisk på www.drvk.dk, ved brug af det allerede tildelte anmeldelsesnummer og adgangskode.

Væsentlige ændringer er bl.a. ændringer, der kan få betydning for forsøgspersonernes sikkerhed, fortolkning af den videnskabelige dokumentation, som projektet bygger på samt gennemførelsen eller ledelsen af projektet. Det kan fx være ændringer i in- og eksklusionskriterier, forsøgsdesign, antal forsøgspersoner, forsøgsprocedurer, behandlingsvarighed, effektparametre, ændringer om de forsøgsansvarlige eller forsøgssteder samt indholdsmæssige ændringer i det skriftlige informationsmateriale til forsøgspersonerne.

Hvor nye oplysninger betyder, at forskeren overvejer at ændre proceduren eller stoppe forsøget, skal komiteen orienteres om det.

Bivirkninger og hændelser

Løbende indberetning

Komiteen skal omgående underrettes, hvis der under projektet optræder formodet alvorlige, uventede bivirkninger eller alvorlige hændelser, jf. komitélovens § 30, stk. 1. Indberetningen skal ledsages af kommentarer om eventuelle konsekvenser for forsøget. Det er kun bivirkninger og hændelser forekommet i Danmark, der skal indberettes. Underretning skal ske senest 7 dage efter, at sponsor eller den forsøgsansvarlige har fået kendskab til tilfældet.

Ved indberetning kan anvendes et skema, der findes på www.nvk.dk. Skemaet med bilag indsendes elektronisk.

Årlig indberetning

Én gang årligt i hele forsøgsperioden skal komiteen have tilsendt en liste over alle formodet alvorlige (ventede og uventede) bivirkninger og alvorlige hændelser, som er indtruffet i forsøgsperioden sammen med en rapport om forsøgspersonernes sikkerhed, jf. komitélovens § 30, stk. 2.

Materialet skal være på dansk eller engelsk.

Ved indberetning skal anvendes et skema, der findes på www.nvk.dk. Skemaet med bilag indsendes elektronisk.

Afslutning
Den forsøgsansvarlige skal senest 90 dage efter afslutningen af projektet underrette komiteen herom, jf. komitélovens § 31, stk. 1. Projektet regnes som afsluttet, når sidste forsøgsperson er afsluttet.

Ved indberetning skal anvendes et skema, der findes på www.nvk.dk. Skemaet indsendes elektronisk.

Afbrydes projektet tidligere end planlagt, skal en begrundelse herfor sendes til komiteen senest 15 dage efter, at beslutningen er truffet, jf. komitélovens § 31, stk. 2.

Hvis projektet ikke påbegyndes, skal dette samt årsagen hertil meddeles komiteen.

Komiteen beder om kopi af den afsluttende forskningsrapport eller publikation, jf. Komitélovens § 28, stk. 2. Vi skal i den forbindelse gøre opmærksom på, at der er pligt til at offentliggøre både negative, positive og inkonklusive forsøgsresultater, jf. komitélovens § 20, stk. 1, nr. 8.

Tilsyn
Komiteen fører tilsyn med, at projektet udføres i overensstemmelse med godkendelsen, jf. komitélovens §§ 28 og 29.

Venlig hilsen

Trine Ziebell
Sekretær i Sekretariatet for den Videnskabsetiske Komité for Region Sjælland

Asiakirja on sähköisesti allekirjoitettu asianhallintajärjestelmässä. Pirkanmaan hyvinvointialue 15.08.2024 klo 15:19. Allekirjoituksen oikeellisuuden voi todentaa kirjaamosta.

Salassa pidettävä

§ 229

7/2024, R24048 Launonen Antti, uusi tutkimus, 2. käsittely, esittelijä Pauniaho Satu-Liisa

12876/2024

Liitteet

1 7_2024_R24048_Launonen_Antti_uusi_tutkimussuunnitelma_2.käsittelyn_materiaalit.pdf
(Salassa pidettävä)
2 6_2024_R24048 Launonen Antti uusi tutkimussuunnitelma_1.käsittelyn_materiaalit.pdf
(Salassa pidettävä)

Esittelyteksti:

Toimikunnan käsiteltäväksi on toimitettu uusi tutkimussuunnitelma toimikunnan 11.6.2024 kokoukseen. Toimikunta pyysi tuolloin lisäselvityksiä ja palautti lausuntohakemuksen uudelleen valmisteltavaksi. Asia on nyt 2. käsittelyssä.

Tutkimuksen nimi:

Leikkaus verrattuna konservatiiviseen hoitoon olkaluun yläosan murtumissa 50-65 vuotiailla potilailla. Pragmaattinen, satunnaistettu, etenevä tutkimus.

Toimeksiantaja:

professori Stig Brorson, sbror@regionsjaelland.dk,
Køgen university hopital,
Lykkebækvej 1, 4600 Køge, Tanska

Tutkimuspaikat ja tutkijat:

TAYS, johtava tutkija dos. Antti Launonen, LL Bakir Sumrein, LL Laura Kärnä, tutkimushoitajat Janika Pietilä, Marketta Rautanen

Køge University Hopital, Køge, Tanska, Line Houkjær, Zaid Issa, Kenneth Brian Holtz ja Stig Brorson

Protokollanumero: 14.6.2024, versio 1.1

Suunniteltu tutkimusaika: 1.9.2024-31.12.2029

Tutkittavien lukumäärä: 30 Pirhassa (koko tutkimus 60)

Laskutustiedot: -

Asiakirja on sähköisesti allekirjoitettu asianhallintajärjestelmässä. Pirkanmaan hyvinvointialue 15.08.2024 klo 15:19. Allekirjoituksen oikeellisuuden voi todentaa kirjaamosta.

Toimitettu materiaali:

-Pirkanmaan hyvinvointialueen alueellisen lääketieteellisen tutkimuseettisen toimikunnan pöytäkirjan ote 6/2024, 185§, kokouspäivämäärä 11.6.2024

-Saatekirje, Launonen Antti, 14.6.2024

-Tiedote tutkimuksesta, 14.6.2024, v1.1

-Lausuntohakemuslomake, 14.6.2024

-Tietosuojaseloste, 14.6.2024

-Suomenkielinen yhteenveto tutkimussuunnitelmasta, v1.1, 14.6.2024

-Tutkimussuunnitelma, v1.1, 14.6.2024

Lisäksi edelliseen kokouskäsittelyyn 6/2024 toimitettu:

-Saatekirje 20.5.2024

-Johtavan tutkijan eettinen arvio 4.3.2024

-Tutkittavan suostumus 16.4.24, v1.0

-Tutkittavan suostumus seurantaryhmälle 16.4.24, v1.0

-Potilasohje kuntoutukseen 4.3.2024, v1.0

-Antti Launosen ansioluettelo 5.3.24

-Tutkimuksen tietosuojan vaikutustenarviointi 20.5.24

-Hakemus lausuntomaksusta vapauttamiseksi 4.3.2024

Ehdotus

Eettinen toimikunta päättää

1. asiasta kokouskäsittelyssä esittelijän esittelystä

2. lausuntomaksun perimisestä

Päätös

1. **Päätös:** eettinen toimikunta päätti antaa otsikossa mainitusta tutkimuksesta myönteisen lausunnon hakemuksen mukaisesti.

Toimikunta pyytää kuitenkin vielä korjaamaan seuraavat kohdat:

Tietosuojaseloste:

Toimikunta kiinnitti huomiota, että tietosuojaselosteen kohdasta 13b puuttuu rasti kohdasta ”oikeus käsittelyn rajoittamiseen” ja tämä on lisättävä. Lisäksi rasti kohdasta ”

Asiakirja on sähköisesti allekirjoitettu asianhallintajärjestelmässä. Pirkanmaan hyvinvointialue 15.08.2024 klo 15:19. Allekirjoituksen oikeellisuuden voi todentaa kirjaamosta.

ei oikeutta tietojen poistamiseen” tulee vaihtaa kohtaan ”oikeus tietojen poistamiseen”, koska tutkimukseen ei liity sellaisia lakivelvoitteita, joista syntyisi pakottava velvoite tietojen säilyttämiseen.

Korjattu tietosuojaseloste on toimitettava eettiselle toimikunnalle tiedoksi. Korjausten toimittamisen yhteydessä on toimitettava myös erillinen allekirjoitettu saatekirje toimikunnan antamiin korjauspyyntöihin; kirjallinen yksityiskohtainen selvitys siitä, mitä korjauksia asiakirjoihin on tehty. Toimikunnan esittämiin korjauspyyntöihin tulee vastata kohta kohdalta.

2. Eettinen toimikunta päätti periä lausuntomaksun: 0 €

Mikäli hakija on tyytymätön saamaansa päätökseen, hänellä on oikeus tehdä siitä oikaisuvaatimus liitteenä olevan oikaisuvaatimusohjeen mukaisesti.

Lisätietoja tutkimuksen esittelijältä.

Korjausten/selvitysten toimittaminen

Tehtyjen muutosten tulee näkyä selkeästi muutetuissa asiakirjoissa. Lisäykset tekstiin tehdään tummennetulla vinotekstillä ja tekstin poistot joko yliviivaten tai harmaapohjalla. Tekstiin tehtyjä muutoksia, lisättyä tai poistettua tekstiä, ei saa merkitä sivujen marginaaliin.

Lausuntomaksu

Ei lausuntomaksua

Esittelijä

Pauniaho Satu-Liisa

Yhteystiedot

Pirkanmaan hyvinvointialueen tutkimuspalvelut

Pirkanmaan hyvinvointialueen alueellinen lääketieteellinen tutkimuseettinen toimikunta

Asiakirja on sähköisesti allekirjoitettu asianhallintajärjestelmässä. Pirkanmaan hyvinvointialue 15.08.2024 klo 15:19. Allekirjoituksen oikeellisuuden voi todentaa kirjaamosta.

PL 2000, 33521 Tampere

toimikunta.eettinen@pirha.fi

Huom. Yhteydenotoissa on ilmoitettava aina ETL-koodi.

Tiedoksi
antti.launonen@pirha.fi; satu.yla-mononen@pirha.fi

Asiakirja on sähköisesti allekirjoitettu asianhallintajärjestelmässä. Pirkanmaan hyvinvointialue 15.08.2024 klo 15:19. Allekirjoituksen oikeellisuuden voi todentaa kirjaamosta.

Otteen oikeaksi todistaa

Tampere
16.08.2024

Minna Lahtinen
TUTKIMUSKOORDINAATTORI

Tiedoksianto asianosaiselle
Päätös on lähetetty sähköpostitse 16.8.2024.

Asiakirja on sähköisesti allekirjoitettu asianhallintajärjestelmässä. Pirkanmaan hyvinvointialue 15.08.2024 klo 15:19. Allekirjoituksen oikeellisuuden voi todentaa kirjaamosta.

Oikaisuvaatimus

§229

Oikaisuvaatimusohje

Oikaisua saa hakea se, johon päätös on kohdistettu tai jonka oikeuteen, velvollisuuteen tai etuun päätös välittömästi vaikuttaa (asianosainen). Oikaisuvaatimus on tehtävä kirjallisesti. Kirjelmässä on ilmoitettava päätös, johon vaaditaan oikaisua, sekä se, millaista oikaisua vaaditaan ja millä perusteilla sitä vaaditaan. Oikaisuvaatimus on toimitettava ennen oikaisuvaatimusajan päättymistä.

Oikaisuvaatimusaika

Oikaisuvaatimus on tehtävä 30 päivän kuluessa päätöksen tiedoksisaannista. Vastaanottajan katsotaan saaneen asiasta tiedon, jollei muuta näytetä, seitsemäntenä päivänä kirjeen lähettämisestä, kolmen päivän kuluttua sähköpostin lähettämisestä tai saantitodistuksen osoittamana aikana tai erilliseen tiedoksisaantitodistukseen merkittynä aikana. Tiedoksiantopäivää ei lueta määräaikaan. Jos määräajan viimeinen päivä on pyhäpäivä tai muu sellainen päivä, jona virastot ovat suljettuna, asiakirjat saa toimittaa ensimmäisenä arkipäivänä sen jälkeen.

A. Oikaisuvaatimus lääketieteellisestä tutkimuksesta annetun lain nojalla annetusta lausunnosta

Eettisen toimikunnan lääketieteellisestä tutkimuksesta annetun lain (488/1999) nojalla antamaan lausuntoon voi vaatia oikaisua. Oikaisuvaatimus tehdään lääketieteellisen tutkimuksen eettisen arvioinnin muutoksenhakujaostolle sähköisen asioinnin kautta osoitteessa https://tukija.fi/oikaisuvaatimuksen-toimittaminen. Sähköposti: info@tukija.fi, Postiosoite: Muutoksenhakujaosto, Tukija, Valvira, PL 43, 00521 Helsinki, Käyntiosoite: Ratapihantie 9, 00520 Helsinki.

B. Oikaisuvaatimus lausuntomaksua koskevaan päätökseen

Alueellisen lääketieteellisen tutkimuseettisen toimikunnan antamaan lausuntomaksua koskevaan päätökseen voi vaatia oikaisua. Oikaisuvaatimus tehdään Pirkanmaan hyvinvointialueen alueelliselle lääketieteelliselle tutkimuseettiselle toimikunnalle. Sähköposti: kirjaamo@pirha.fi, Postiosoite: Pirkanmaan hyvinvointialue, Kirjaamo, PL 272, 33101 Tampere, Käyntiosoite: Biokatu 12, 33520 Tampere.

C. Ihmisen elimien, kudoksien ja solujen lääketieteellisestä käytöstä annetun lain nojalla annettu kielteinen lausunto

Jos alueellisen lääketieteellisen tutkimuseettisen toimikunnan ihmisen elimien, kudoksien ja solujen lääketieteellisestä käytöstä annetun lain (101/2001, kudoslaki) 11 §:n 1 momentin 1 kohdan, 19 §:n 2 tai 3 momentin taikka 20 §:n 1 momentin nojalla antama lausunto on kielteinen, Lääkealan turvallisuus- ja kehittämiskeskus Fimea tekee hakemuksesta asiassa päätöksen. Sähköposti: kirjaamo@fimea.fi, Postiosoite: PL 55, 00034 FIMEA.

D. Kliinisten lääketutkimusten muutoksesta annettu kielteinen lausunto

Asiakirja on sähköisesti allekirjoitettu asianhallintajärjestelmässä. Pirkanmaan hyvinvointialue 15.08.2024 klo 15:19. Allekirjoituksen oikeellisuuden voi todentaa kirjaamosta.

Alueellisen lääketieteellisen tutkimuseettisen toimikunnan lausuntoon ei voi vaatia oikaisua eikä siitä voi valittaa. Tutkimuksen toimeksiantaja voi saattaa asian uudelleen asianomaisen alueellisen lääketieteellisen tutkimuseettisen toimikunnan käsiteltäväksi. Asianomaisen alueellisen lääketieteellisen tutkimuseettisen toimikunnan on tällöin toimeksiantajan pyynnöstä hankittava asiasta ennen uuden lausuntonsa antamista valtakunnallisen lääketieteellisen tutkimuseettisen toimikunnan (Tukija) lausunto.

E. Oikaisuvaatimus kliinisestä laitetutkimuksesta annetusta kielteisestä lausunnosta

Alueellisen lääketieteellisen tutkimuseettisen toimikunnan lääkinnällisistä laitteista annetun lain (719/2021) nojalla antamasta kielteisestä lausunnosta ei voi vaatia oikaisua eikä siitä voi valittaa.

F. Oikaisuvaatimus biopankkitutkimuksesta annettuun lausuntoon

Alueellisen lääketieteellisen tutkimuseettisen toimikunnan lausuntoon, joka on annettu lääketieteellisestä tutkimuksesta annetun lain (488/1999) sekä biopankkilain (688/2012) nojalla, voi vaatia oikaisua. Oikaisua ei voi vaatia mikäli lausunto on annettu yksin biopankkilain (688/2012) nojalla. Oikaisuvaatimus tehdään lääketieteellisen tutkimuksen eettisen arvioinnin muutoksenhakujaostolle sähköisen asioinnin kautta osoitteessa https://tukija.fi/oikaisuvaatimuksen-toimittaminen. Sähköposti: info@tukija.fi, Postiosoite: Muutoksenhakujaosto, Tukija, Valvira, PL 43, 00521 Helsinki, Käyntiosoite: Ratapihantie 9, 00520 Helsinki.

G. Näytteiden siirto biopankkiin – kielteinen lausunto

Alueellisen lääketieteellisen tutkimuseettisen toimikunnan lausuntoon ei voi vaatia oikaisua eikä siitä voi valittaa. Jos eettinen toimikunta ei pidä näytteiden siirtoa biopankkiin eettisesti hyväksyttävänä, Lääkealan turvallisuus- ja kehittämiskeskus Fimea tekee hakemuksesta asiasta päätöksen. Sähköposti: kirjaamo@fimea.fi, Postiosoite: PL 55, 00034 FIMEA.

Aika 13.8.2024, klo 12:00 - 14:50

Paikka Teams etäkokous

Läsnä olleet jäsenet

Matti Korppi, puheenjohtaja
Kaija Puura, läsnä 227–231§, 235–241§, 243–254§, varapuheenjohtaja
Janne Aittoniemi
Annika Auranen, läsnä 219–246§, 248–254§
Eeva Boman
Anna Heikkinen
Merja Helenius
Juuso Kuusisto
Outi Laine
Lauri Lehtimäki
Elina Mattila
Teemu Murtola, läsnä 219–224§, 233–234§, 242§
Erja-Leena Paukkeri, läsnä 226–232§, 235–241§, 243–254§
Satu-Liisa Pauniaho
Virpi Marjukka Tiainen
Vesa Virtanen
Johanna Palmio
Arvi Yli-Hankala
Eija Kasari, varajäsen
Johanna Leimola, varajäsen
Henri Toivanen, varajäsen

Muut läsnä olleet

Kirsi Kohonen, sihteeri
Minna Lahtinen, sihteeri
Elina Ellilä, sihteeri

Poissa

Sauli Palmu
Teemu Paarlahti

Supplementary Information, File 7:

Salary guarantees and funding documentation

List of funding sources for the study "Surgical versus non-surgical treatment of proximal humerus fracture in patients aged 50–65 years: Young shoulder CARE (displaCed proximAl humerRus fractueE) trial – a pragmatic randomized controlled trial study protocol":

The study has received partial funding from:

- **Region Zealand Health Science Research Foundation** [Region Sjællands Sundhedsvidenskabelige Forskningsfond] (Ref. R41-A1846), amount: 186,542 DKK, dated 19 January 2024.
- **Spies Foundation**, amount: 25,000 DKK, dated 24 April 2024.
- **Lippmann Foundation**, amount: 65,000 DKK, dated 14 May 2024.

Confirmation letters are available in Danish:

Fra: Region Sjælland <no-reply@efond.dk>
Sendt: 19. januar 2024 08:37
Til: Line Løjbert Houkjær; Forskningsstøtte Center
Emne: Region Sjælland Sundhedsvidenskabelige Forskningsfond - A1846 Stig Brorson

Forsigtig: Ekstern mail
Kære Stig Brorson

Vedr. ansøgning nr. R41-A1846 om forskningsmidler fra Region Sjællands Sundhedsvidenskabelige Forskningsfond (RSSF) 2024:

Projekttitel: 'Surgical versus non-surgical treatment in adults aged 50 to 65 years with a displaced proximal humerus fracture: a protocol for a pragmatic randomized controlled trial'

Fondsbestyrelsen for RSSF har behandlet din ansøgning og har truffet afgørelse om at støtte projektet med kr. 186.542.

Bevillingen kan kun bruges til det ansøgte projekt med det beskrevne formål.

Bevillingen overføres til sygehuset ved den kommende budgetoverførsel og administreres af sygehusets økonomikontor. Du bør tage kontakt til sygehusets økonomikontor inden 14 dage og oplyse om den modtagne projektbevilling. Sygehusets økonomikontor opretter en projektkonto, hvor projektets udgifter konteres. Projektet vil indgå i afdelingens budget. Projektets udgifter skal godkendes af afdelingsledelsen før fremsendelse til kontering i økonomikontoret. Der henvises i øvrigt til regionens kasse- og regnskabsregulativ.

Ved årsskifte har afdelingsledelsen og økonomikontoret ansvar for, at overføre saldoen på projektet til næste års budget.

Ved afslutning af projektet har afdelingsledelsen og økonomikontoret ansvar for at uforbrugte midler tilbageføres til Forskningsstøtte Center, Region Sjælland. Dette gøres i forbindelse med årsafslutningen. Ved afslutning af projektet skal slutrapport udfyldes via efond www.efond.dk/rsj

Har du spørgsmål til bevillingen, er du velkommen til at henvende dig på e-mail:
forskninngsstotte@regionsjaelland.dk

Region Sjælland tilbyder support til at finde og søge forskningsmidler. Du kan tage kontakt til

Region Sjællands Fondskontor på fondskontor@regionsjaelland.dk.

Med venlig hilsen

Bo Borg Mikkelsen
Chefkonsulent, Forskningsstøtte Center, Region Sjælland

2024

Center for evidensbaseret ortopædkirurgi, Sjællands Universitets Hospital, Køge.

CVR NR. 29190658

TILDELES HERMED:

Kr. 25.000,-

TIL NEDENSTÅENDE FORMÅL:

Behandling af overarmsbrud hos 50-65 årige

Beløbet er bevilliget efter ansøgning hos Simon Spies Fonden.
Ansøgningen er behandlet og vurderet af fondens bestyrelse.

På vegne af Simon Spies Fonden ønskes held og lykke med formålet.

JANNI SPIES, FORMAND
Simon Spies Fonden

SIMON SPIES FONDEN

Fra: Anne Mette Bruun <mbr@rglaw.dk>
Sendt: 14. maj 2024 13:25
Til: Line Løjbert Houkjær
Cc: Marie Raz Haargaard
Emne: VS: Lippmann Fonden

Forsigtig: Ekstern mail
Line Houkjær
lihol@regionsjaelland.dk

Deres ansøgning har den 6. maj 2024 været behandlet på et bestyrelsesmøde i Lippmann Fonden. Bestyrelsen har besluttet at tildele Dem en legatportion på kr. 65.000 som støtte til projektet "Kirurgisk eller ikke-kirurgisk behandling af forskudte brud på den øverste del af overarmen i aldersgruppen 50-65 år".
De bedes pr. mail (mbr@rglaw.dk) meddele mig Deres CVR/CPR nr. samt bankkonto, hvortil beløbet ønskes overført.

Det tildelte beløb udbetales under forudsætning af, at det ansøgte projekt gennemføres. Desuden skal beløbet anvendes inden for 2 år fra bevillingsdatoen. I modsat fald bortfalder tildelingen.

Med venlig hilsen

Anne Mette Bruun
Fondschef

Rovsing & Gammeljord
Poul Ankers Gade 2, 2. tv.
DK-1271 Copenhagen K

T +45 53 73 71 60
D +45 53 7831 67

mbr@rglaw.dk

www.rglaw.dk
